# Supplementary material for: The nanoscale molecular morphology of docked exocytic dense-core vesicles in neuroendocrine cells
Source: Nat Commun. 2021 Jun 25;12:3970. doi: 10.1038/s41467-021-24167-9 (PMC8233335; doi:10.1038/s41467-021-24167-9)
Supplement: Supplementary file 1 — Supplementary Information [file 41467_2021_24167_MOESM1_ESM.pdf]

Supplementary Information

The nanoscale molecular morphology of docked exocytic dense-core vesicles in neuroendocrine cells

Bijeta Prasai[1], Gideon J. Haber[1], Marie-Paule Strub[1], Regina Ahn[1], John A. Ciemniecki[1], Kem A. Sochacki[1], Justin W. Taraska[1]*

1. Biochemistry and Biophysics Center, National Heart, Lung, and Blood Institute, National Institutes of Health, Bethesda, MD 20892
*Correspondence should be addressed to Justin W. Taraska (justin.taraska@nih.gov).

Supplementary Figure 1

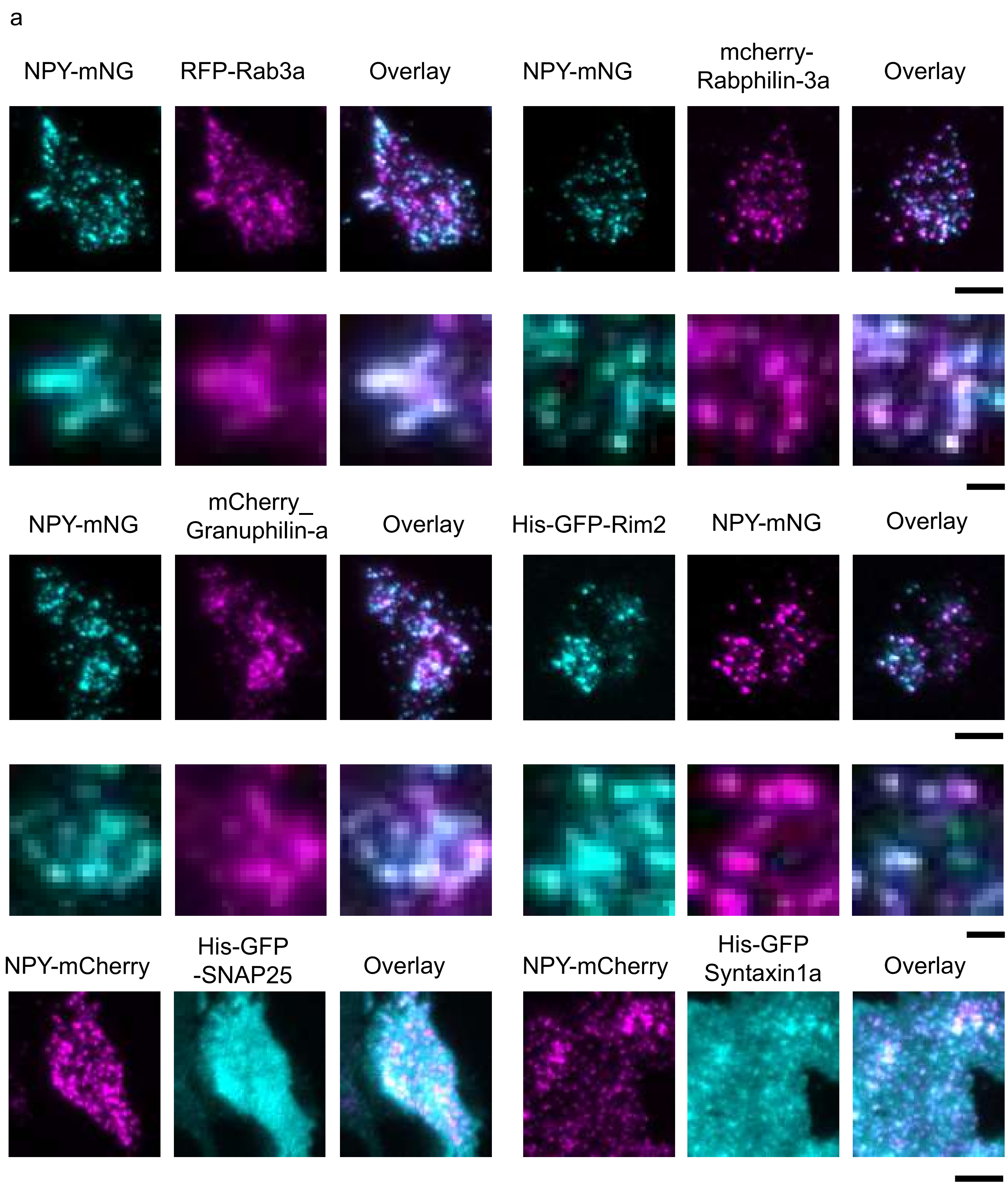

b

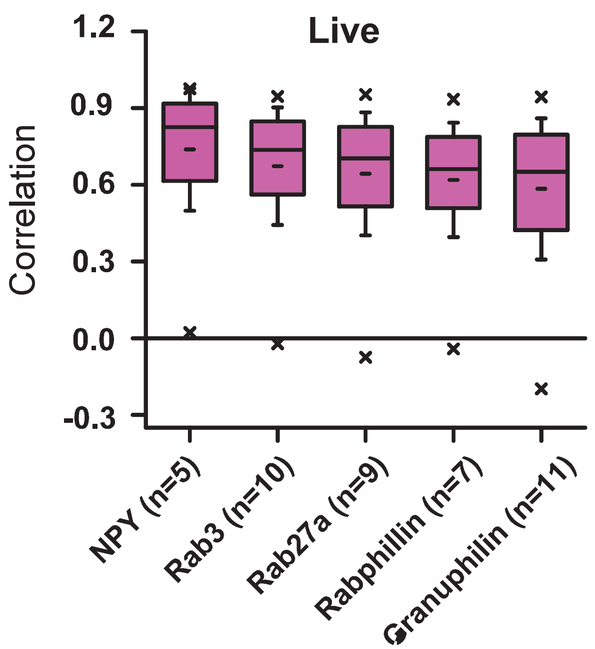

Supplementary Figure 1. Colocalization of proteins and vesicles in unroofed and live cells. (a) TIRF microscopy images of unroofed and fixed PC12 cells co-transfected with mNG or mCherry labeled NPY, and mRFP or mCherry-labeled Rab3a, Rabphilin3a, Granuphilin-a, and Rim2, and their overlay. Scale bar is 5 μm for whole cell images (upper panel) and 1 μm for zoomed in images (lower panel). TIRF microscopy images of unroofed and fixed PC12 cells expressing His-GFP-Syntaxin1A and His-GFP-SNAP25 and co-transfected with NPY-mCherry, and their overlay are shown in lower most row (scale bar=5 μm). (b) Correlation analysis for five proteins with NPY-GFP–labeled DCVs in live intact PC12 cells. Cells are sorted based on their mean correlation values. 'n' next to the proteins denotes the number of cells analyzed from one correlative experiment (for both a and b). Boxes (magenta) are the 25th–75th percentile range of data, and the whiskers are the SD. The solid bar is the median, and the small dash is the mean. The × marks above and below each data set are the 1st and 99th percentiles.

Supplementary Figure 2

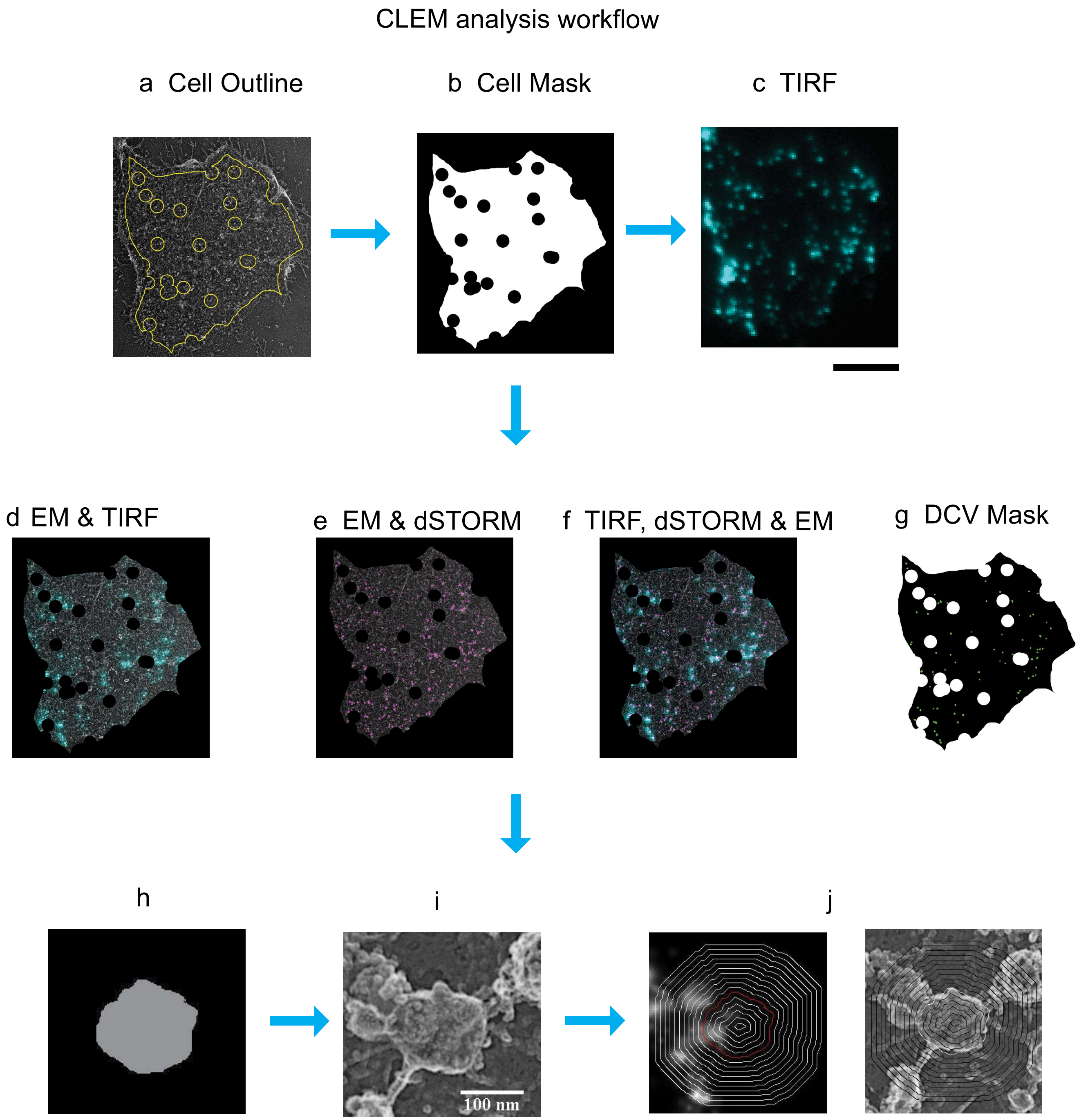

Supplementary Figure 2. CLEM analysis workflow. (a) PREM cell membrane and gold fiducials were outlined. (b) Next, binary cell mask was created that exclude gold fiducials (~1 μm). Cell binary mask was added with (c) TIRF image to obtain (Scale bar = 5 μm) (d) TIRF/EM overlay. Similarly (e) EM/dSTORM or (f) TIRF/dSTORM/EM overlay were created. Dense core vesicles were identified and outlined from either d/e/f and (g) a mask for DCVs generated. Individual vesicles in (h) dSTORM and (i) EM were isolated and (j) segmented from either center or edge. TIRF/STORM/EM data were collected from two independent experiments.

Supplementary Figure 3

a

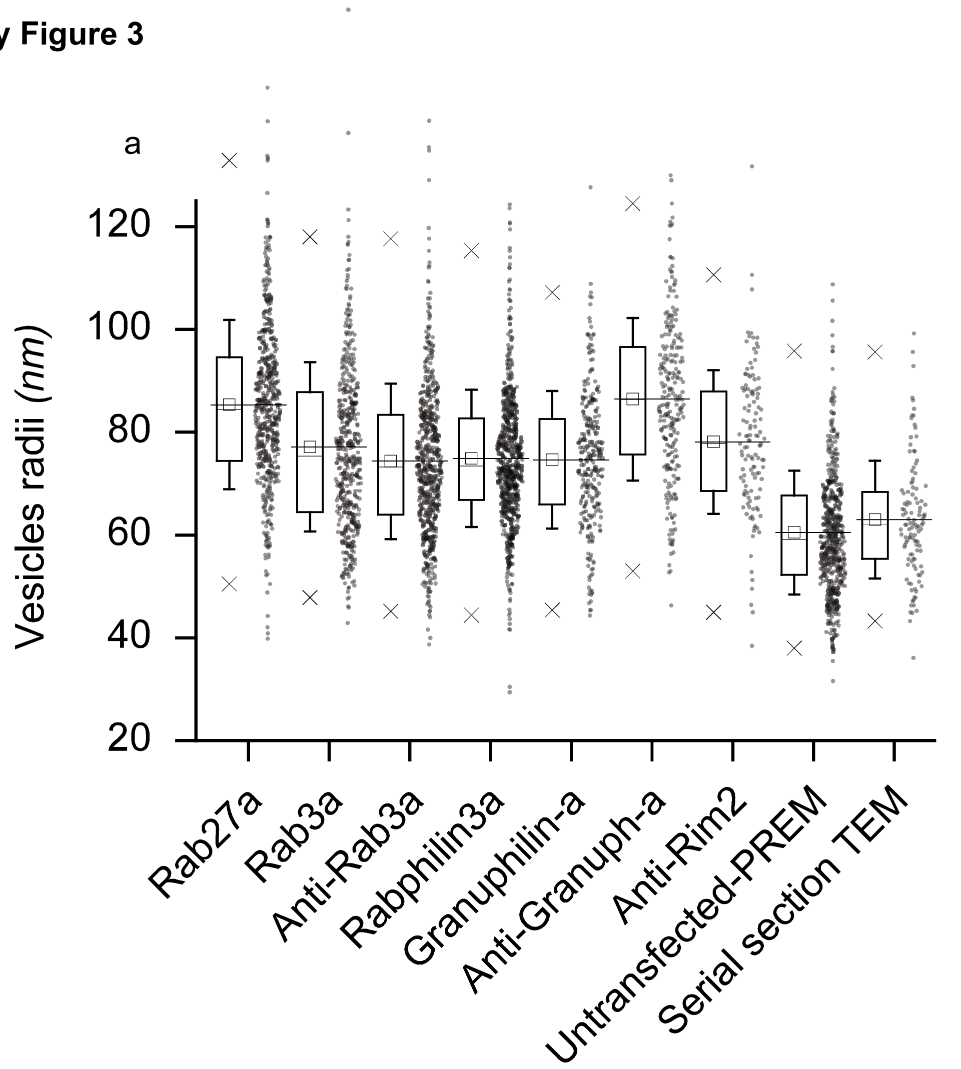

b

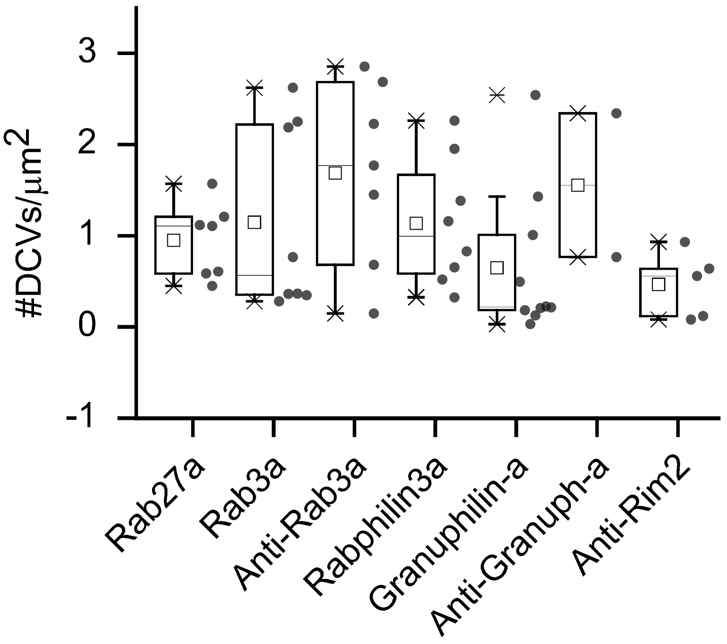

c

PC12- untransfected-PREM

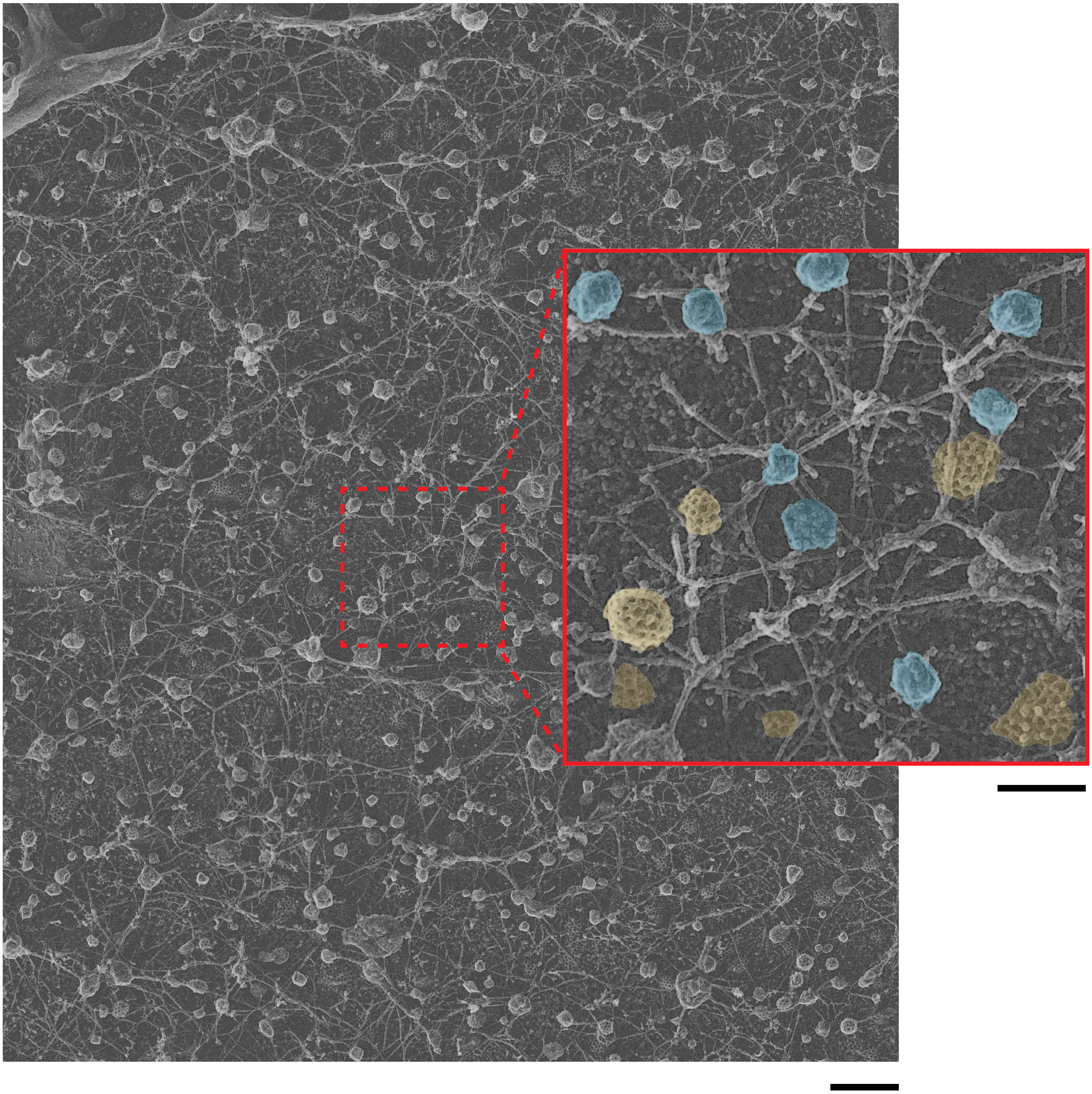

Supplementary Figure 3 Continued

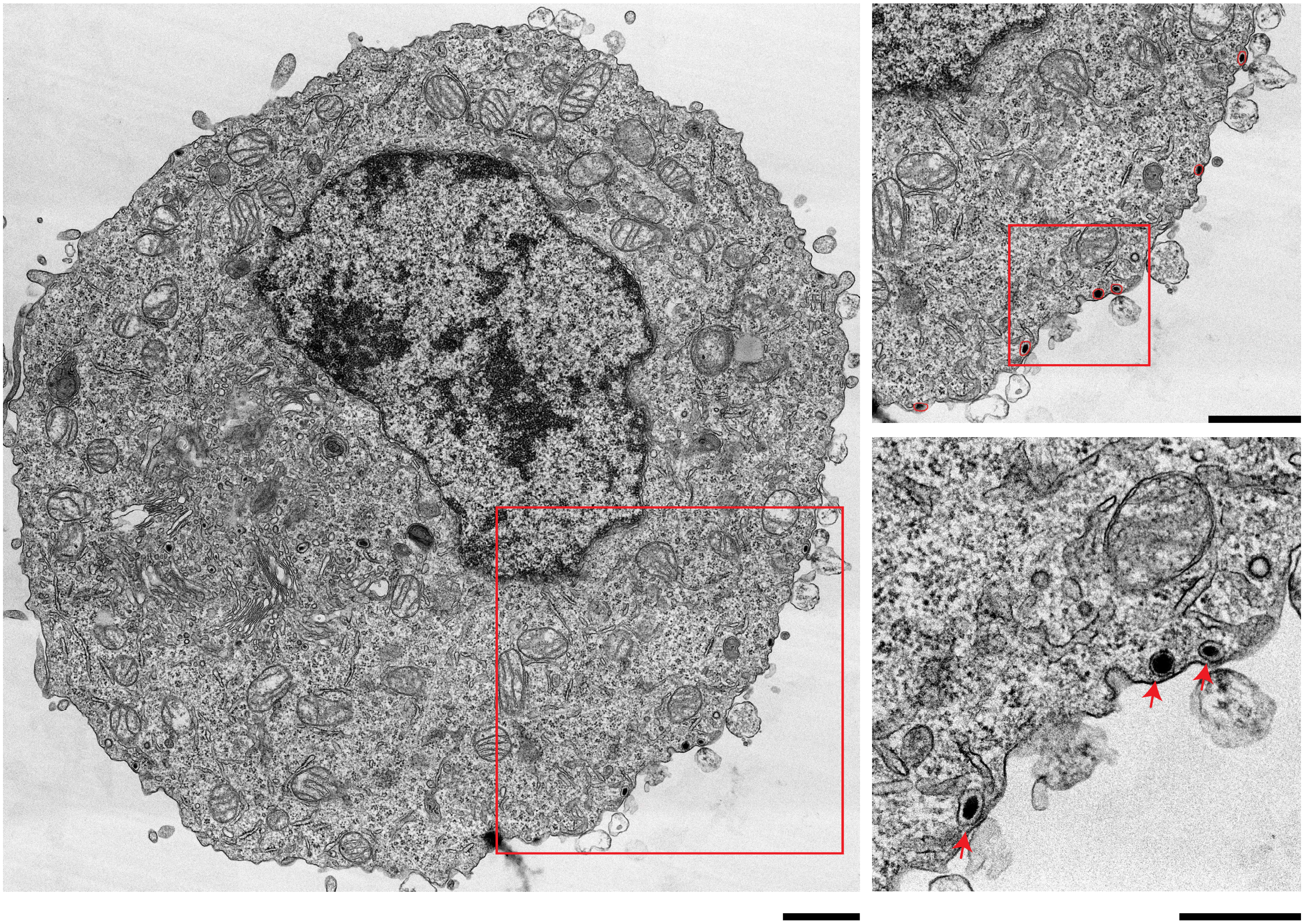

Supplementary Figure 3. Size and density of DCVs measured from PREM and thin-section TEM. (a) Radius of the DCVs measured from PREM images obtained from PC12 cells. (b) The number of DCVs present in squared micron area of PREM membrane. Boxes are the 25th–75th percentile range of data, and the whiskers are the standard deviation. The solid bar is the median, and the small dash is the mean. The × marks above and below each data set are the 1st and 99th percentiles. The number of vesicles used in the radius and density measurements are available in Supplementary Table 1b. dGFP-Rab27a: n = 7 cells; 514 vesicles; dGFP-Rab3a: n = 8 cells, 451 vesicles; anti-Rab3a: n = 7 cells, 581 vesicles; dGFP-Rabphilin3a: n = 8 cells, 674 vesicles; dGFP-Granuphilin-a: n = 10 cells, 284 vesicles; anti-Granuphilin-a: n = 2 cells, 244 vesicles; anti-Rim2: n = 5 cells,126 vesicles; untransfected PC12 (PREM): n = 3 cells, 521 vesicles; untransfected PC12 (ultra-thin TEM): n = 8 cells, 118 vesicles. (c) PREM image of an untransfected PC12 cell (scale bar=500 nm). Enlarged image from red dashed-box shows the difference in the morphology of clathrin coated structures highlighted in yellow and DCVs highlighted in blue (scale bar=200 nm). This morphology was observed in two independent experiments. (d) Ultra-thin TEM section of a whole untransfected PC12 cell (scale bar=1 μm). In the right panel, the upper image (scale bar=500 nm) shows a region of the cell section on left panel outlining (in red) the dense core vesicles. The lower image in the right panel is the magnified area highlighting dense core vesicles (scale bar=500 nm). This morphology was observed in three independent experiments.

Supplementary Figure 3 Continued

Supplementary Figure 4

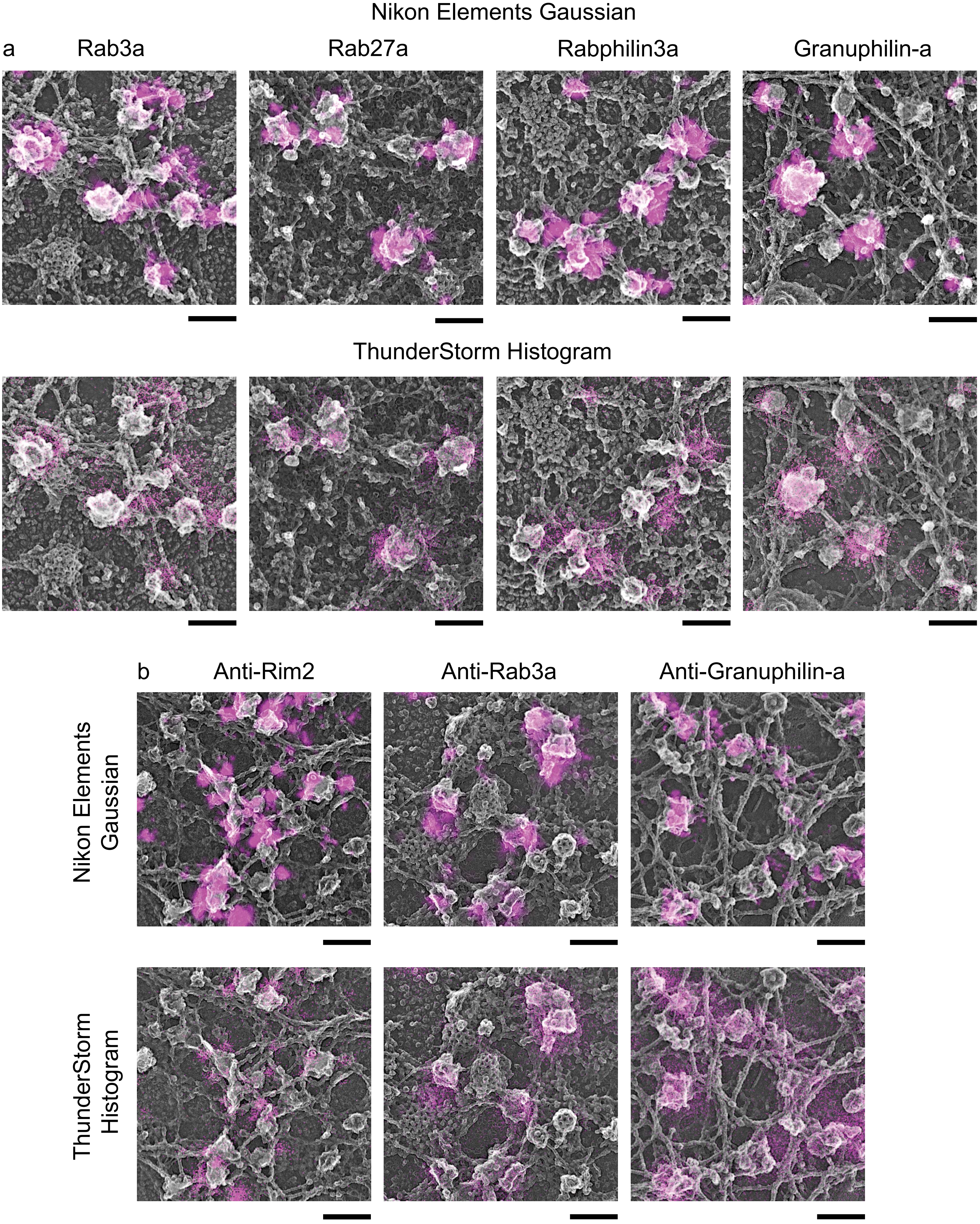

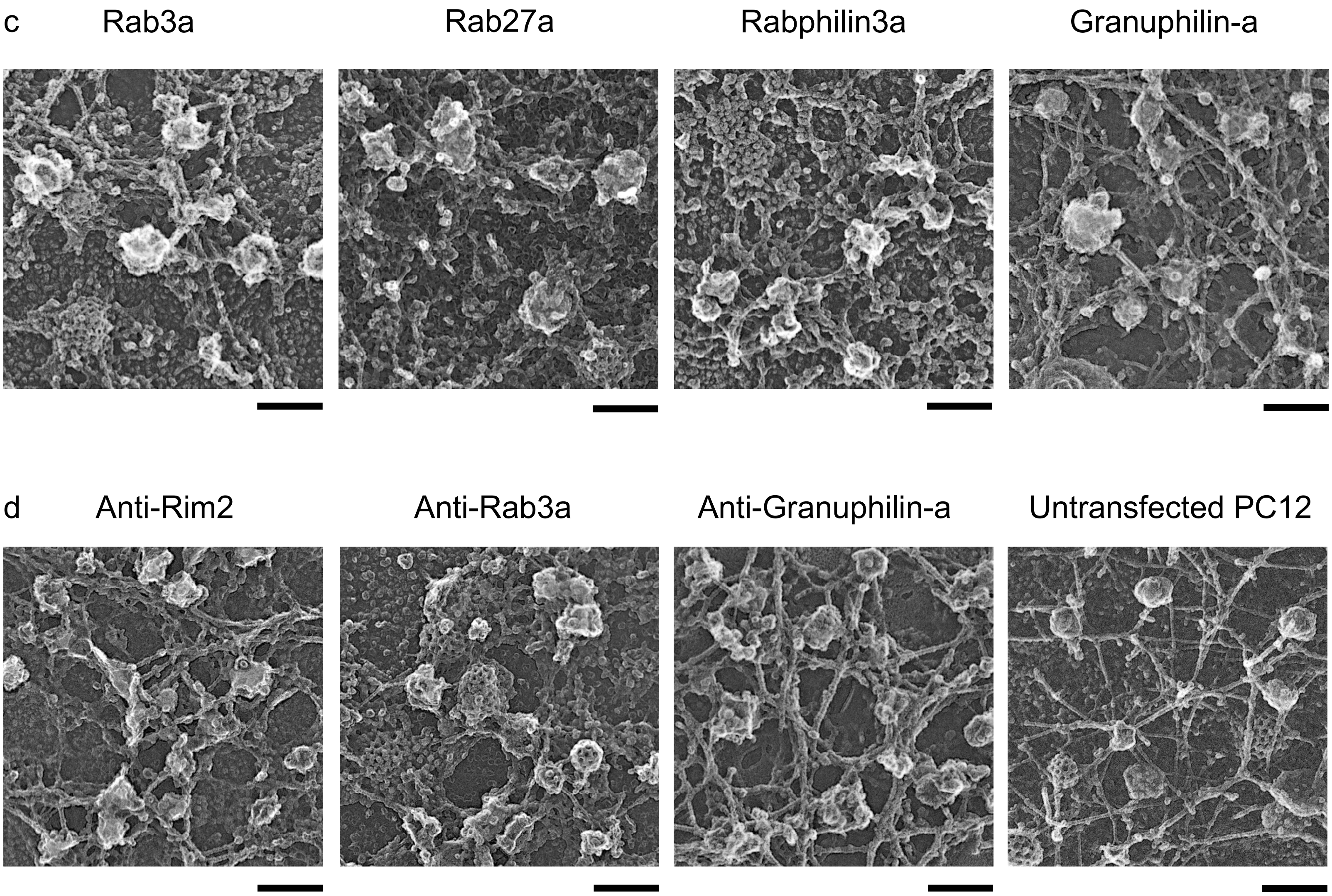

Supplementary Figure 4. CLEM in Figure 2 for Gaussian and histogram dSTORM. CLEM for Gaussian rendered dSTORM images processed with Nikon elements (upper panel, and CLEM for dSTORM images processed with ThunderStorm and visualized as histogram (lower panel) shown for (a) overexpressed proteins, Rab3a, Rab27a, Rabphilin3a, Granuphilin-a, and (b) endogenous proteins, Rim2, Rab3a, and Granuphilin-a. Different photon counts and frame merging parameters are the causes for the more diffuse Gaussians (ThunderStorm, Fig. 2) versus the sharper and more compact spots in images produced with Nikon Elements. (c, d) EM images in the figures a and b shown without overlaying with dSTORM images. The magnified image of the untransfected PC12 cell in supplementary fig. 3c is also shown. Scale bars are 200 nm. Overexpression CLEM data were collected from over two independent experiments and immunolabeled CLEM data from one experiment, PREM image of an untransfected PC12 cell was observed in two and ultra-thin TEM section of untransfected PC12 cell observed in three independent experiments.

Supplementary Figure 4 Continued

Supplementary Figure 5

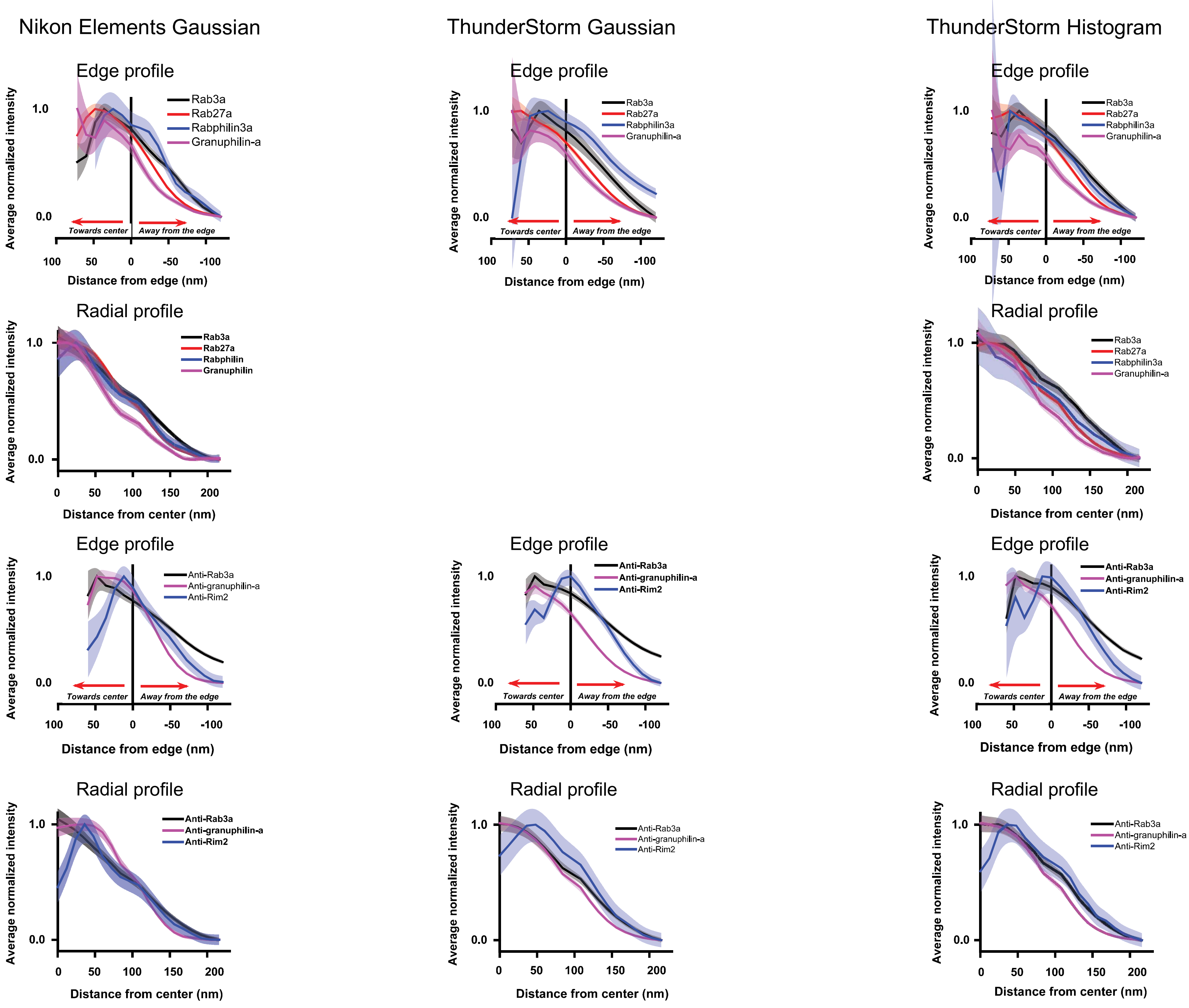

Supplementary Figure 5. Edge and radial fluorescence profiles for expressed and endogenous proteins for ThunderStorm and Nikon Elements processed dSTORM. Edge fluorescence profiles show normalized average fluorescence intensity distribution towards the center and away from the edge of the vesicles for cells expressed with dark GFP fused proteins in the first row. The second row shows radial fluorescence profile for the expressed proteins. dGFP-Rab3a (n = 8 cells; edge: 455 vesicles, radial: 458 vesicles), dGFP-Rab27a (n = 7 cells; edge: 544 vesicles, radial: 545), dGFP-Rabphilin3a (n = 8 cells; edge: 659 vesicles, radial: 644 vesicles), dGFP-Granuphilin-a (n = 10 cells; edge: 285 vesicles, radial: 275 vesicles). The mean fluorescence intensity is shown as a dark line (black=Rab3a; red=Rab27a; blue=Rabphilin3a; magenta=Granuphilin-a). The third and fourth rows respectively show edge and radial fluorescence profiles for immunolabeled endogenous Rab3a (n = 7 cells; edge: 598 vesicles, radial: 596 vesicles), Granuphilin-a (n = 2 cells; edge: 252 vesicles, radial: 252 vesicles), and Rim2 (n = 5 cells; edge: 128 vesicles, radial: 129 vesicles). The mean fluorescence intensity is shown as a dark line (black=anti-Rab3a; magenta=anti-Granuphilin-a; blue=anti-Rim2). The standard error of the mean is shown in transparency. The profiles for dSTORM images processed with Nikon Elements and ThunderStorm imageJ plugin are shown in columns. The first two columns show the profiles generated for Gaussian rendered dSTORM images from the specified software. The third column shows the profiles for histogram representation of dSTORM images processed with ThunderStorm. The number of cells and vesicles analyzed are available in Supplementary Table 1a. Overexpression CLEM data were collected from over two independent experiments and immunolabeled CLEM data from one experiment. The structures without any fluorescence were excluded from the analysis. The slope decrease in the profiles for ThunderStorm processed images when compared to the profiles for the data processed with Nikon Elements can be attributed to the different photon counts and default frame merging parameters used in Nikon Elements. The edge and radial plot profiles obtained from the histogram representation of the images largely resemble the Gaussian profiles.

Supplementary Figure 6

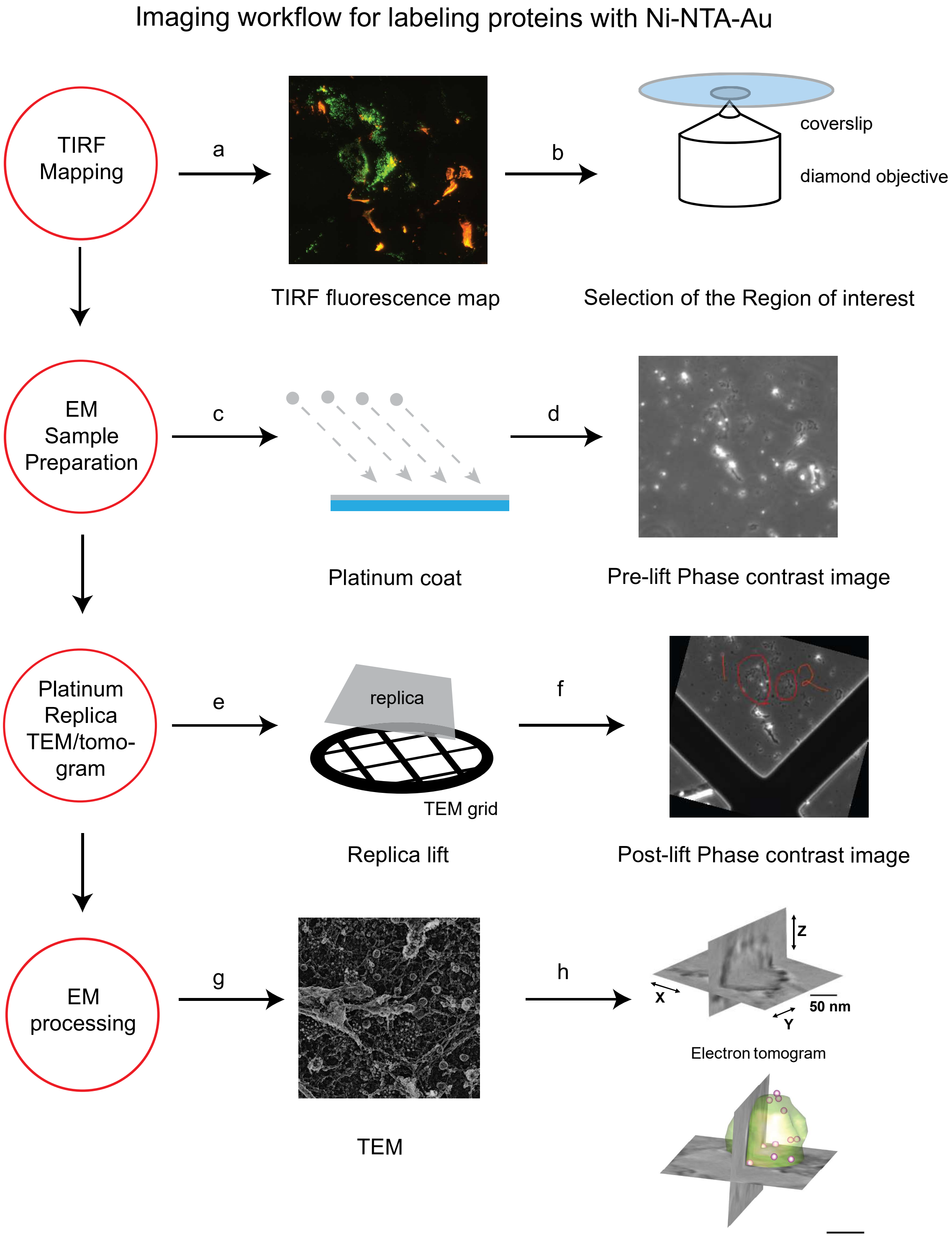

Supplementary Figure 6. Ni-NTA-Au labeled proteins imaging and analysis pipeline. (a) Cells labeled with Ni-NTA-Au were mapped (fluorescence) to obtain a 20 × 20 large montage. (b) The mapped region was marked by etching the coverslip using a diamond objective marker. (c) The sample was then prepared for EM, critical point dried, and coated with platinum and carbon. (d) The region of interest on the coverslip was imaged with 20 × phase-contrast objective. (e) The replica was lifted, transferred to TEM grid, and (f) imaged post-lift for phase-contrast. (g) 2D TEM and (h) electron tomogram of the GFP fluorescent cells acquired with single-axis tilt series (-60˚ to 60˚, at 1˚ increment) using a JEOL1400 TEM and tomograms were reconstructed and processed with IMOD software. Scale bar is 50 nm. Two independent imaging experiments were performed for 2D- and 3D-EM.

Supplementary Figure 7

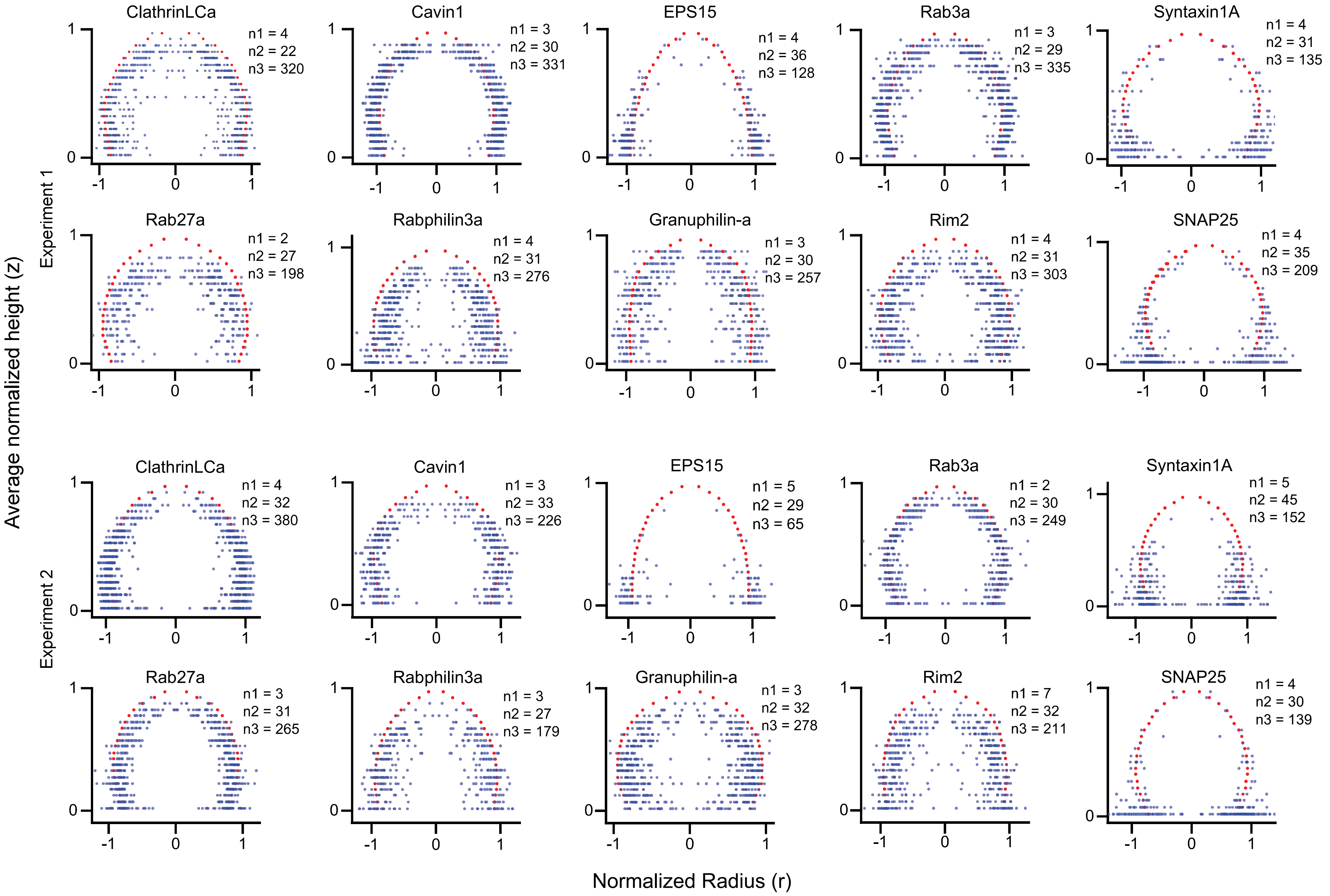

Supplementary Figure 7. Vesicle profiles showing 3D distribution of gold on CCVs and DCVs in two independent experiments. Average vesicle membrane profile and gold nanoparticle distribution for the proteins Clathrin light chain A, Cavin1, EPS15, Rab27a, Rab3a, Rabphilin3a, Granuphilin-a, Rim2, Syntaxin1A, and SNAP25 as seen in two independent experiments. The distribution is presented symmetrically across the ordinate. The notation n1, n2 and n3 refers to the number of tomograms, vesicles, and gold nanoparticles used in the data analysis, respectively.

Supplementary Figure 8

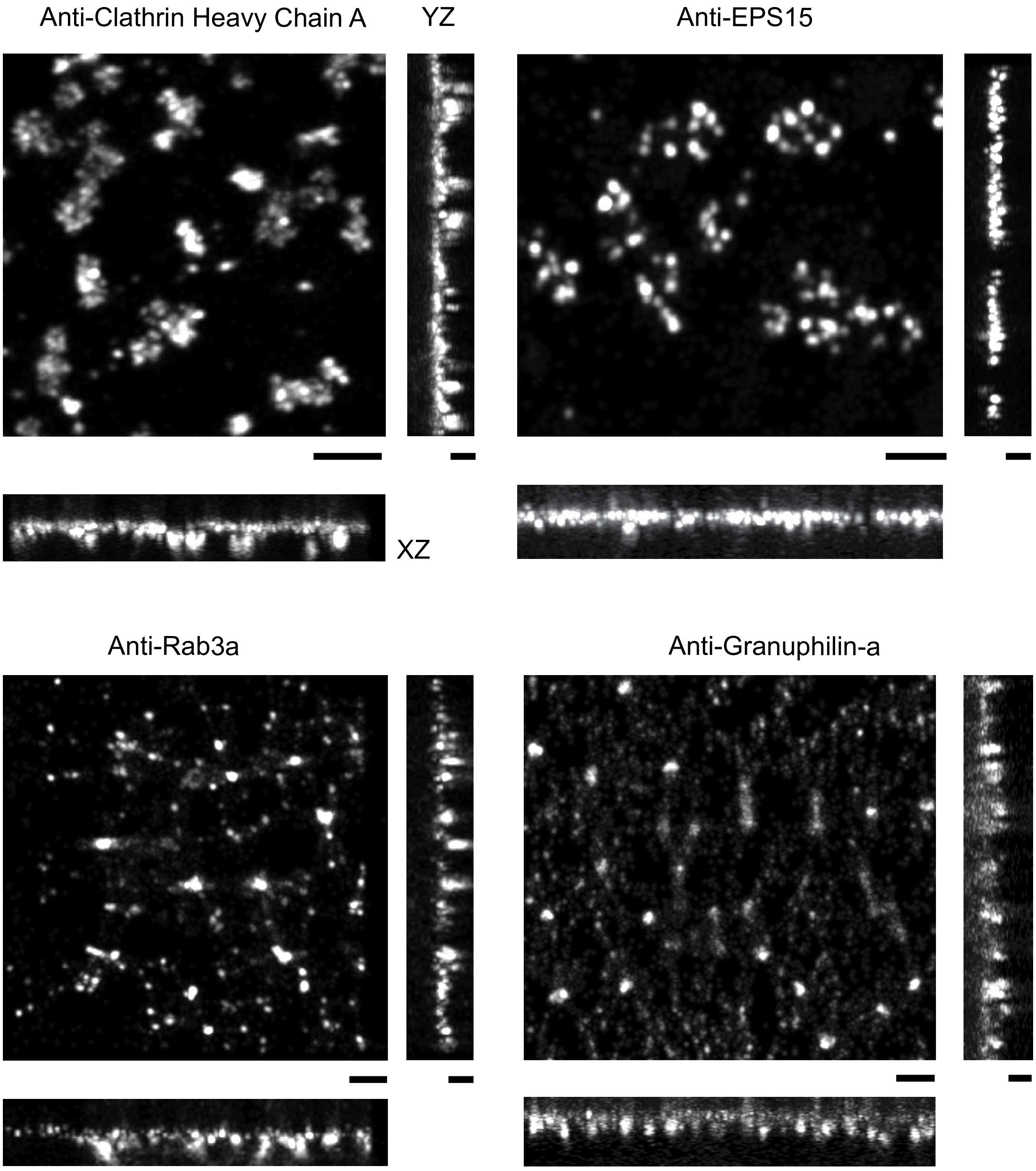

Supplementary Figure 8. 3D-STORM images showing distribution of endogenous proteins on CCVs and DCVs. Cells immunolabeled with anti-clathrin heavy chain (Hela), anti-EPS15 (Hela), anti-Rab3a (PC12), and anti-granuphilin-a (PC12) and fluorescently labeled with Alexa fluor 647 conjugated anti-rabbit or anti-mouse F(ab')2 fragment. The figures show maximum intensity projections of an XY (scale bar=500 nm), XZ and YZ views (scale bar=300 nm). Two independent 3D-STORM imaging experiments were performed.

Supplementary Figure 9

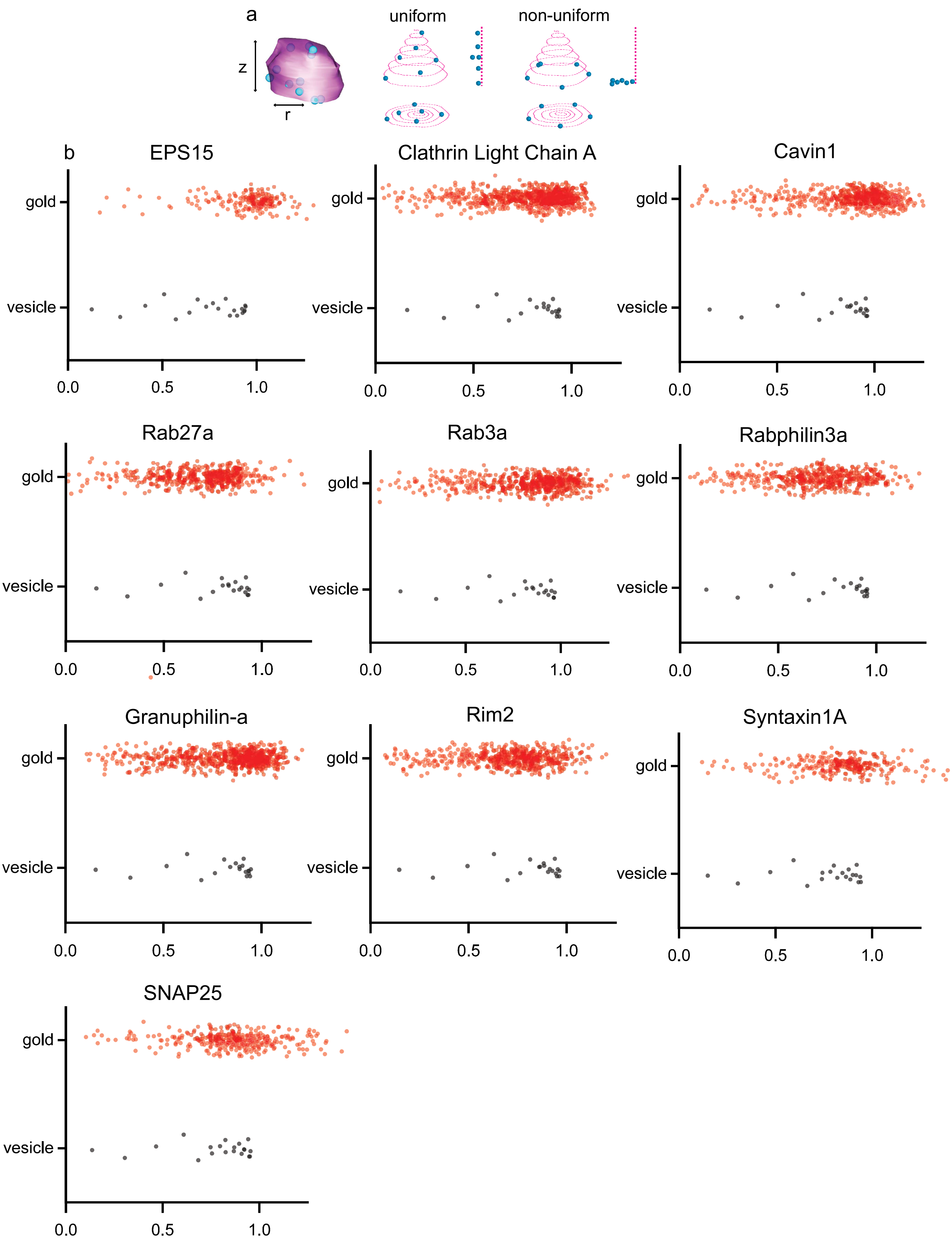

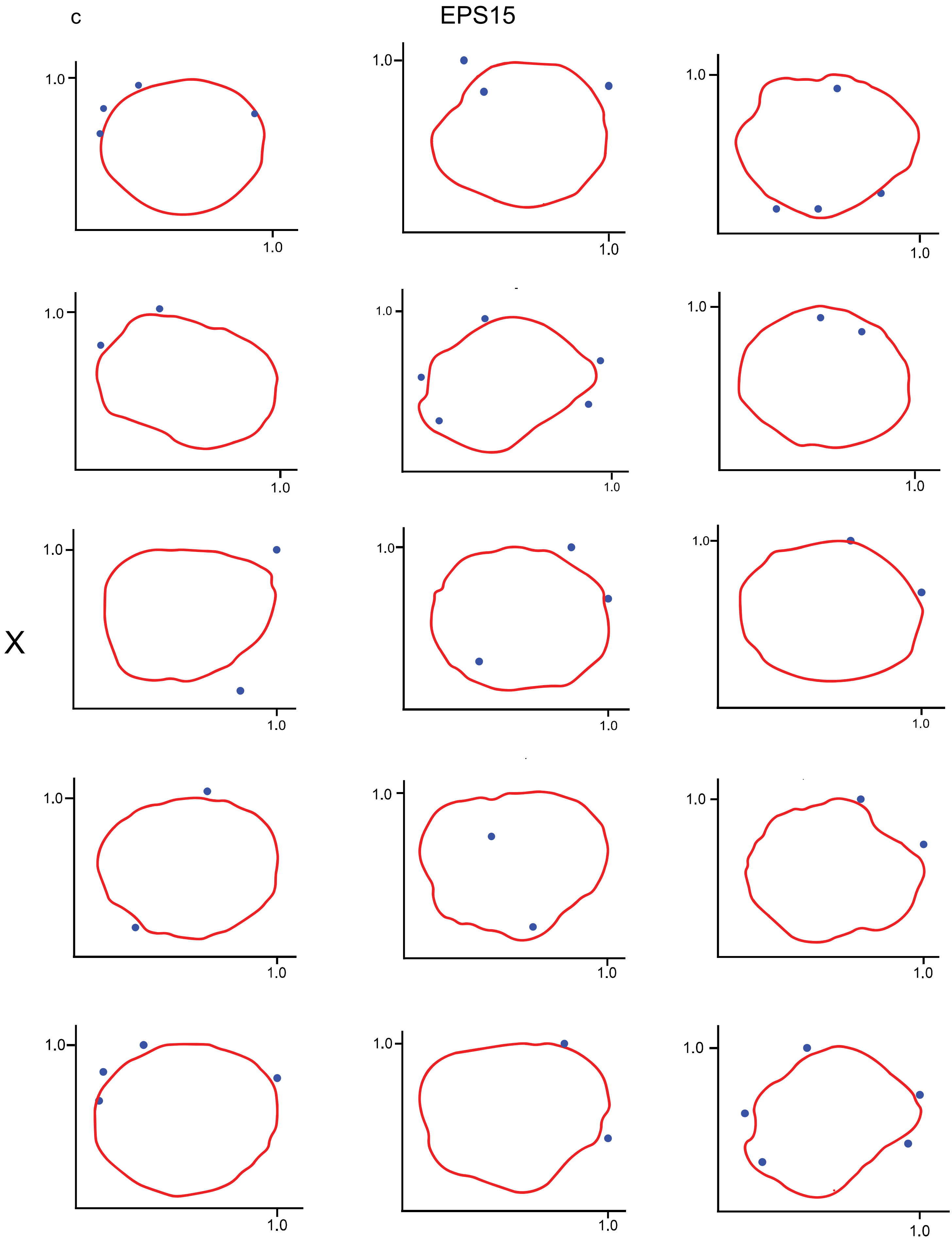

Supplementary Figure 9 Continued

d

ClathrinLight ChainA

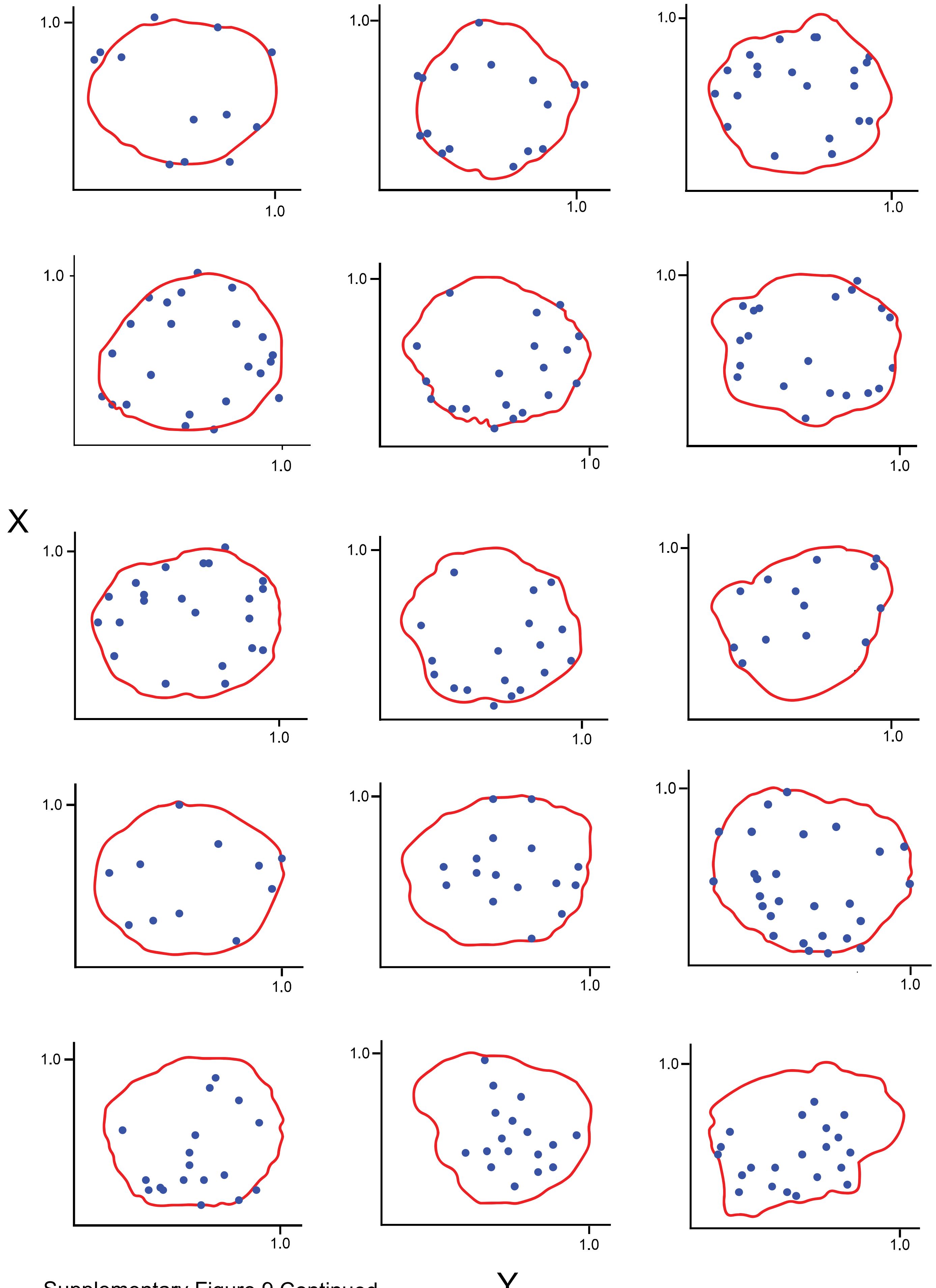

Supplementary Figure 9 Continued

e

Rab3a

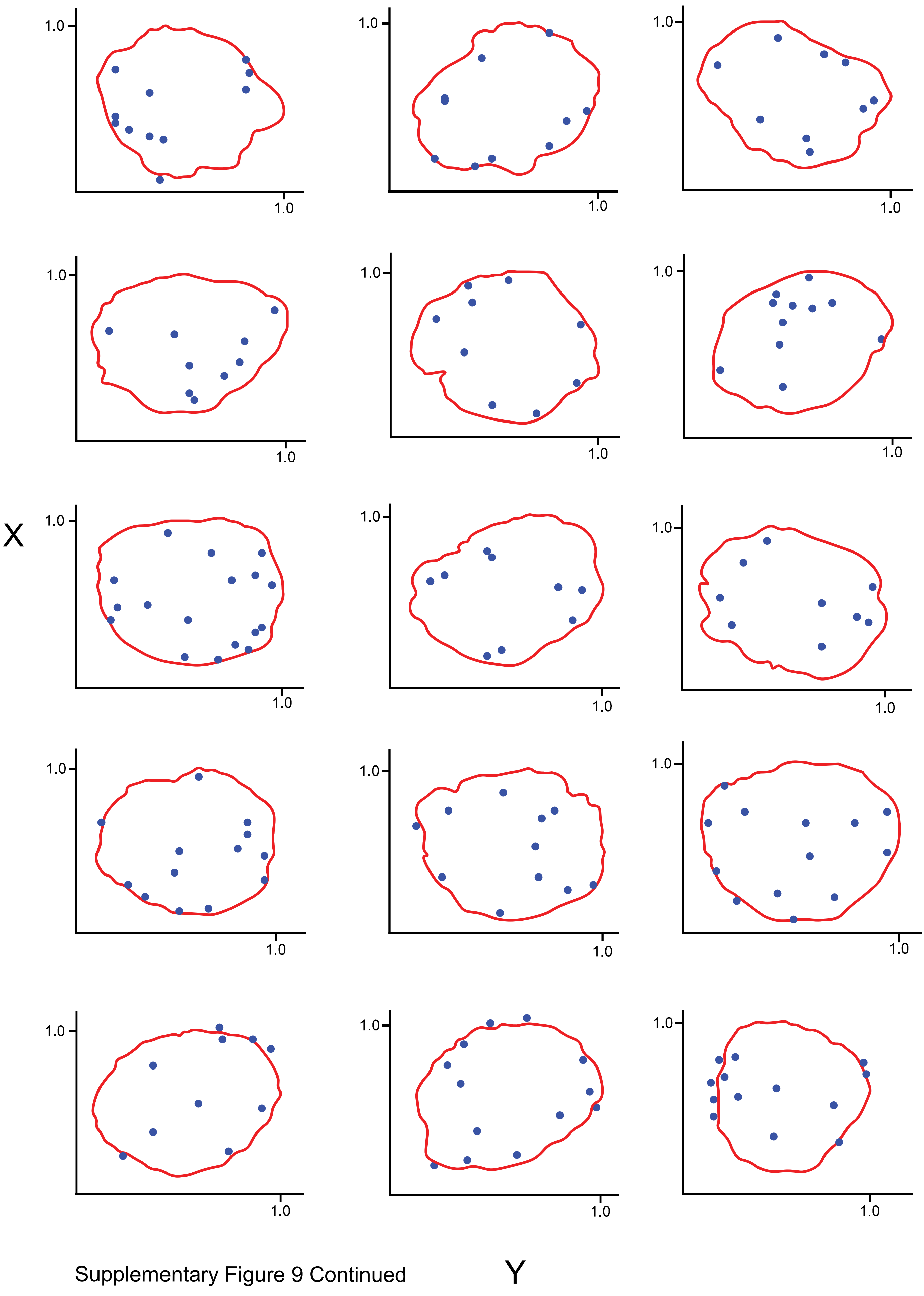

Supplementary Figure 9 Continued

f

SNAP25

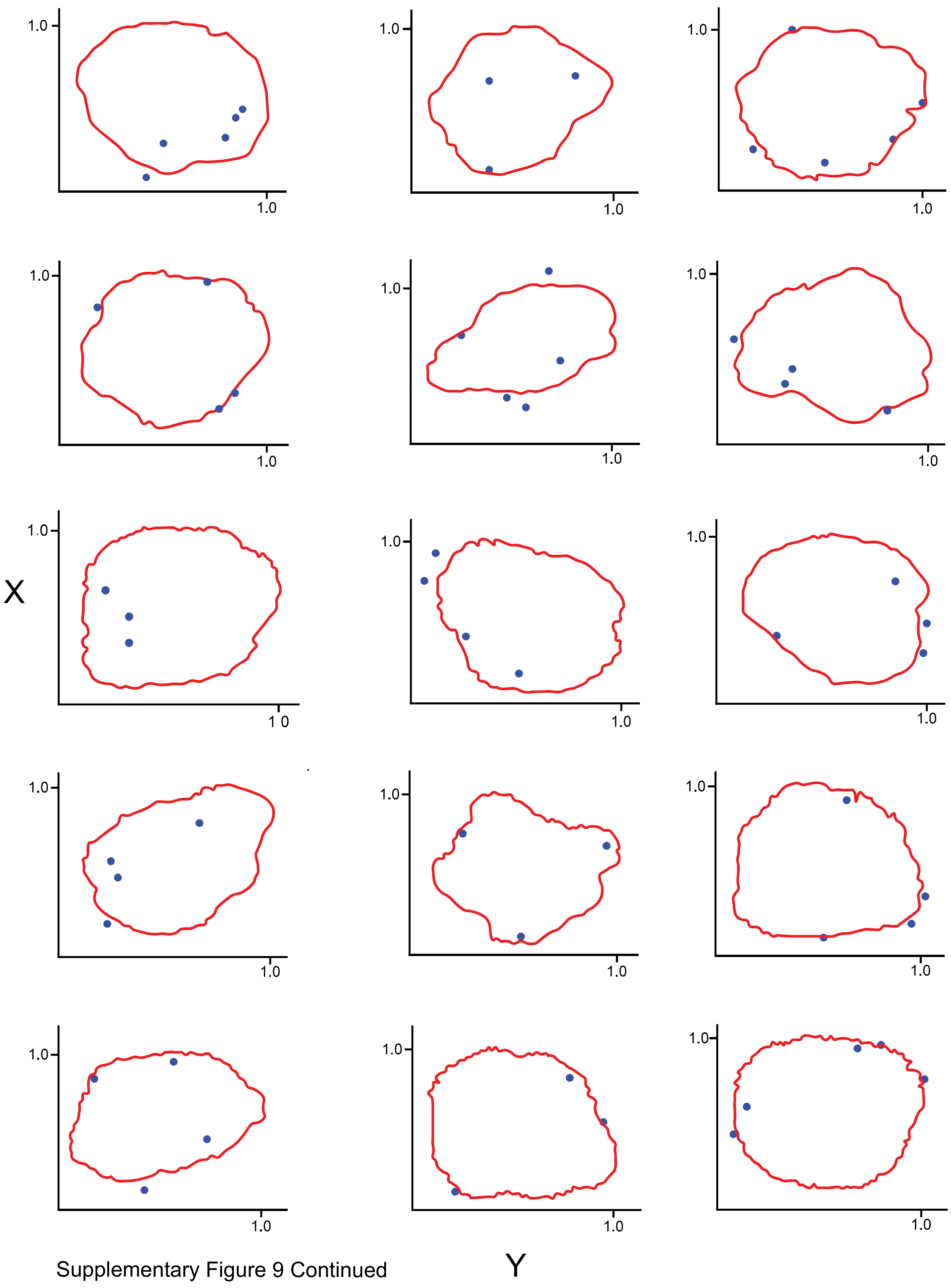

Supplementary Figure 9 Continued

Supplementary Figure 9. Uniform and non-uniform distribution of proteins visualized in one-dimension. (**a**) 3D model view (left panel) of a vesicle from vesicle membrane contour points mesh and gold particles scatter points. Schematics (right panel) showing the contour points (coordinates) of tomogram z slices of a vesicle from top to the base of the structure, and uniform (left) and non-uniform (right) distribution of gold particles on vesicle contour. The right and lower panels in each schematic show how the distribution would look like when compressing the coordinates in one dimension. (**b**) The distribution of gold particles shown for various proteins when the axial dimension of the vesicle model in Figure 5 is compressed. The distribution of gold particles shown for individual vesicles by plotting normalized raw x and y positions of vesicle membrane and gold particles for (**c**) EPS15, (**d**) clathrin light chain A, (**e**) Rab3a, and (**f**) SNAP25. The red outline represents the perimeter traced for the x and y coordinates of the vesicle.

Supplementary Figure 10

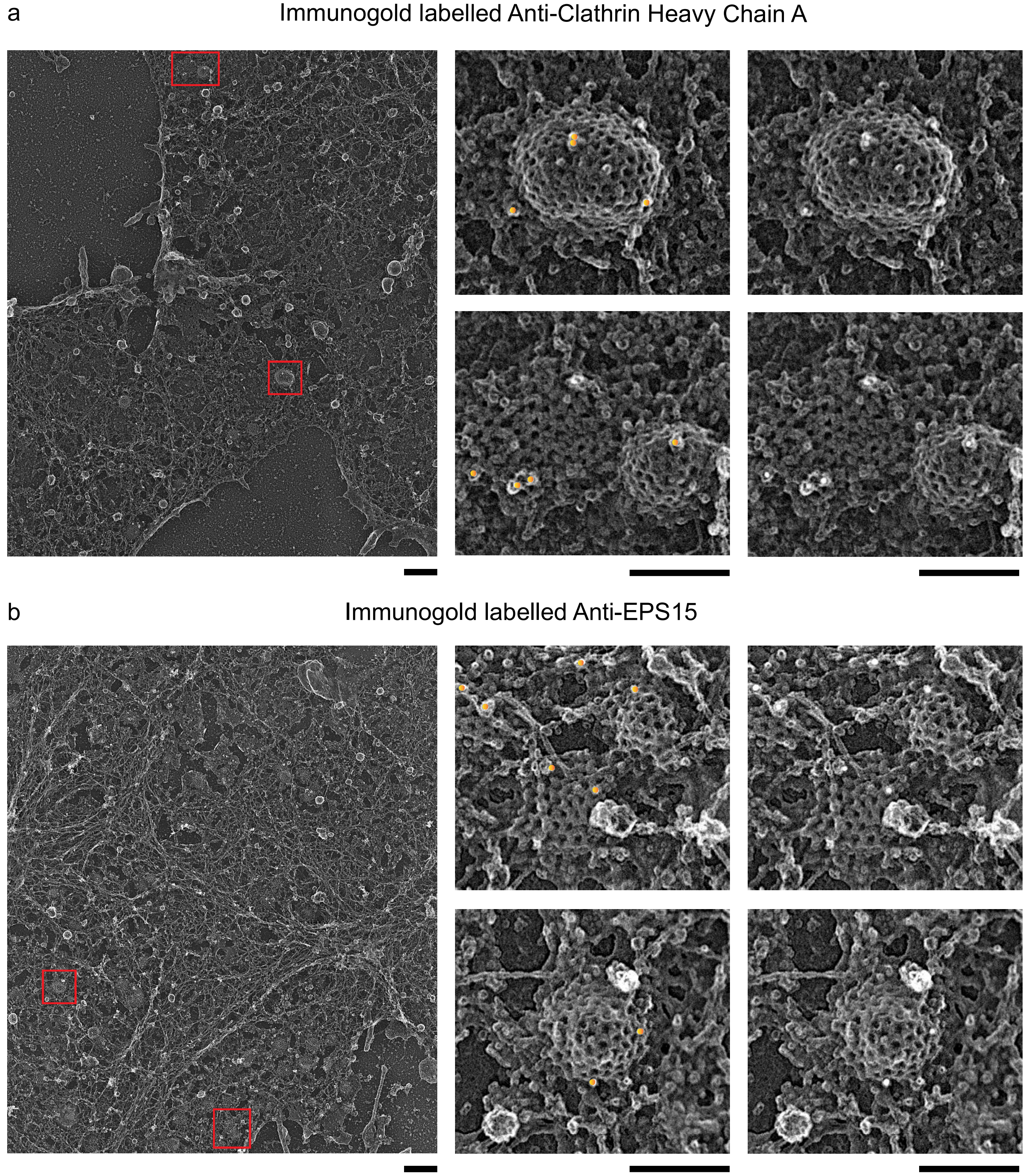

Supplementary Figure 10. PREM images of immunogold labeled clathrin coated vesicles. Hela cells immunolabeled with (a) anti-clathrin heavy chain and (b) anti-EPS15, and labeled with 10 nm secondary immunogold. Left panel shows a large region of PREM images (scale bar=500 nm). Middle panel shows magnified view of individual clathrin coated vesicles from image in left panel (red boxes). Immunogold are marked with orange circles (scale bar=200 nm). Right panel shows the vesicles in middle panel without the orange markings (scale bar=200 nm). One immunogold labeled EM imaging experiment was performed.

Supplementary Figure 11

dGFP-Rab3a (Large image for crops in Fig. 2)

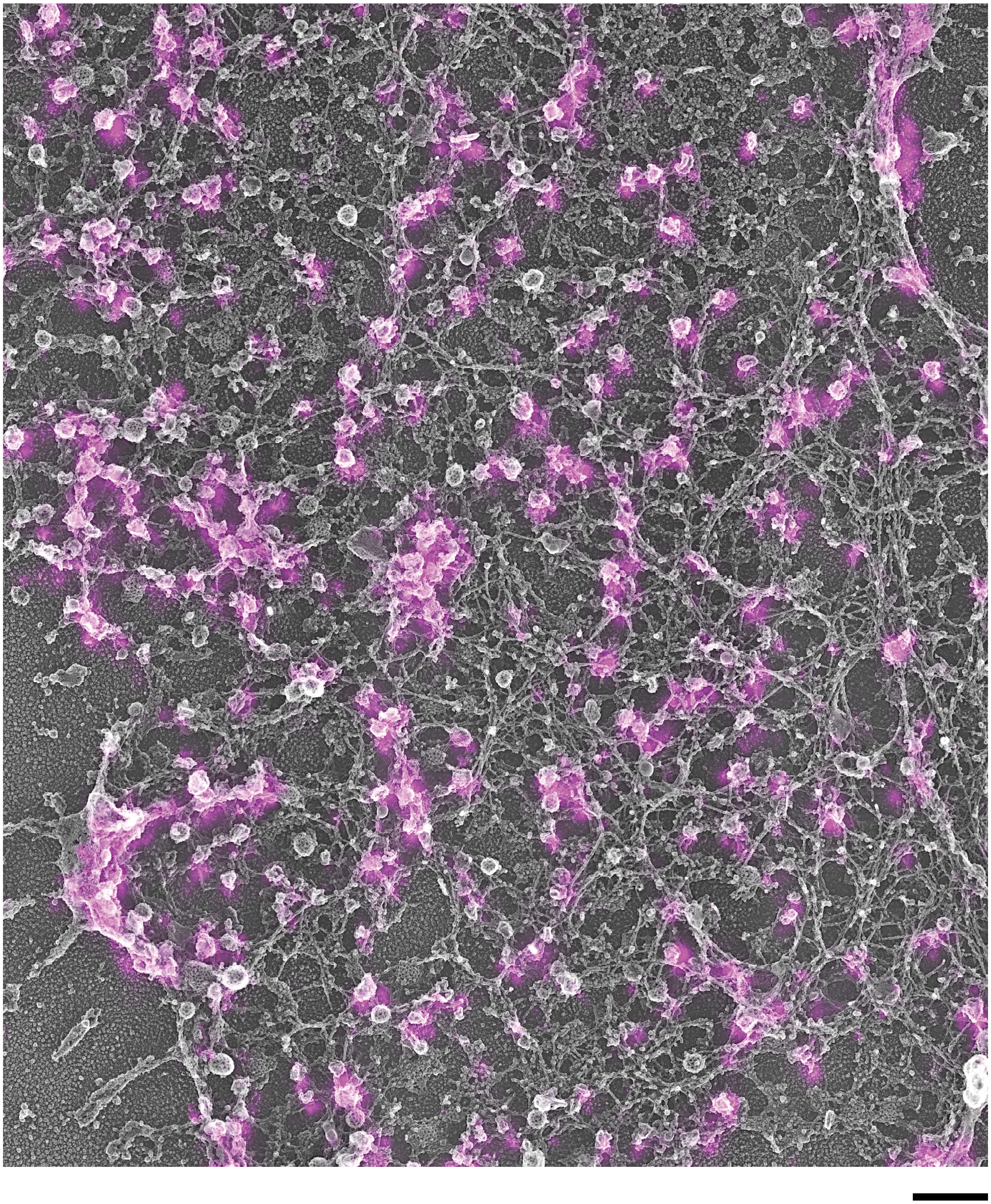

dGFP-Rab27a (Large image for crops in Fig. 2)

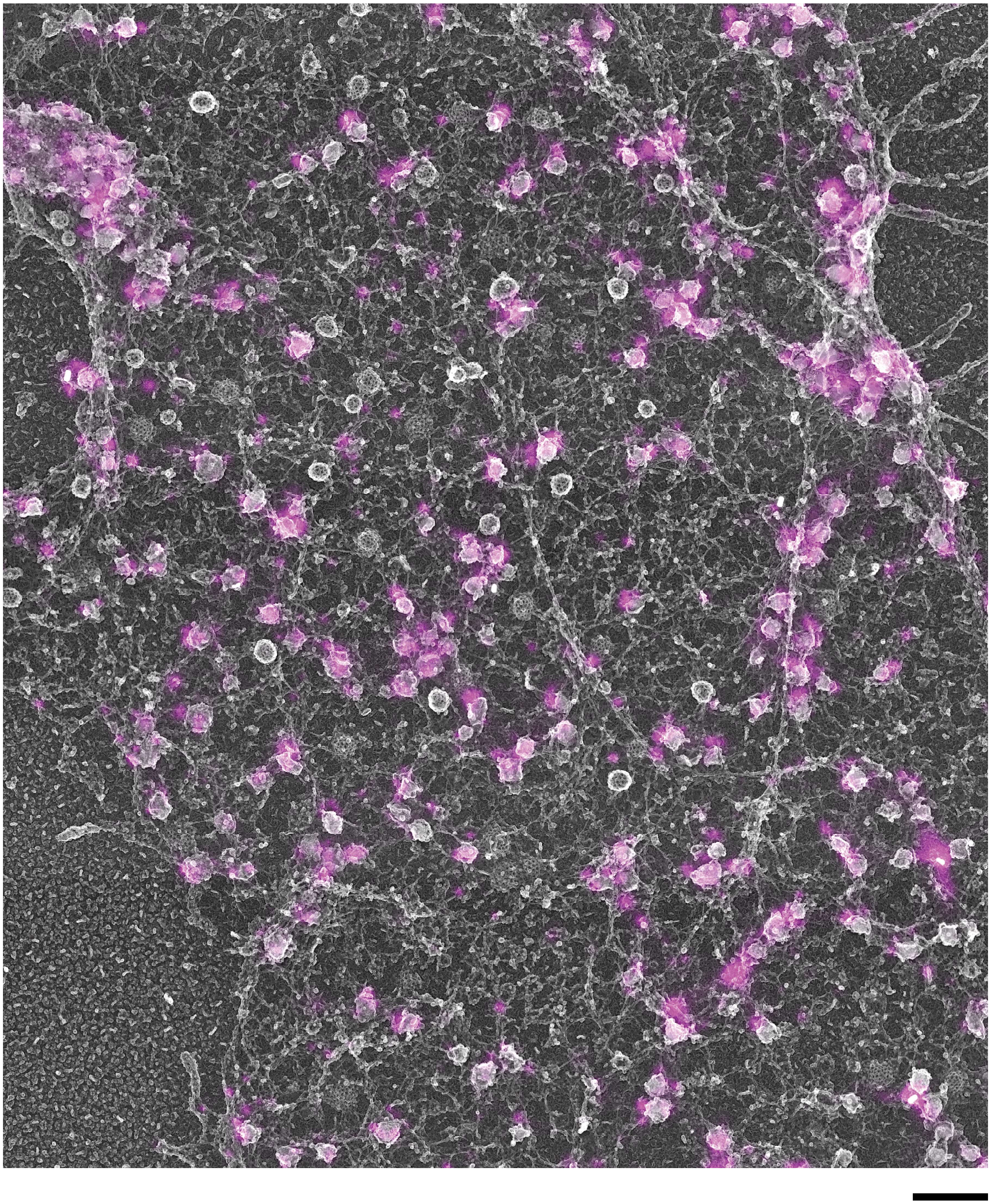

Supplementary Figure 11 Continued

dGFP-Rabphilin3a (Large image for crops in Fig 2)

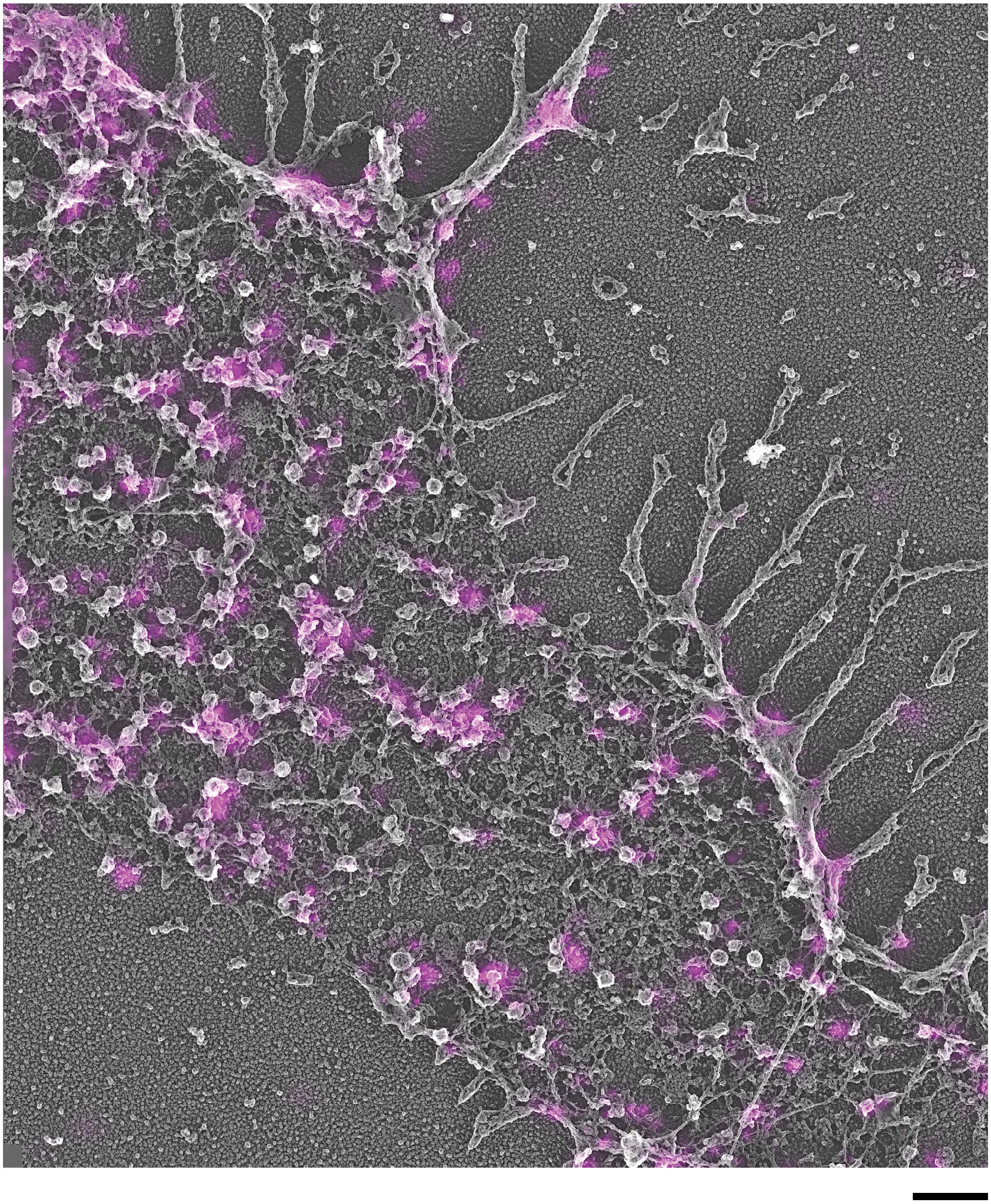

Supplementary Figure 11 Continued

dGFP-Granuphilin-a (Large image for crops in Fig 2)

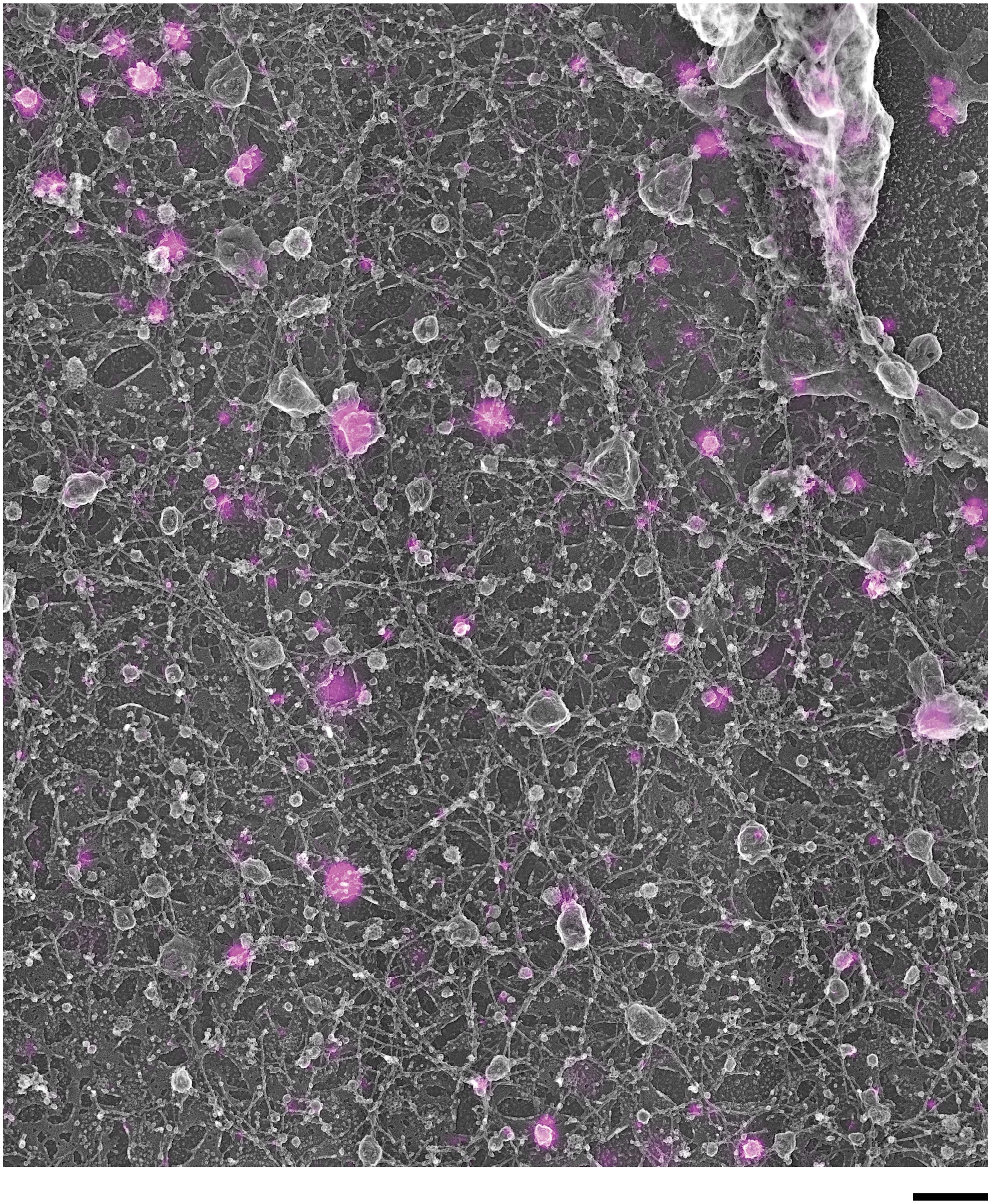

Supplementary Figure 11 Continued

Anti-Rim2 (Large image for crops in Fig 2)

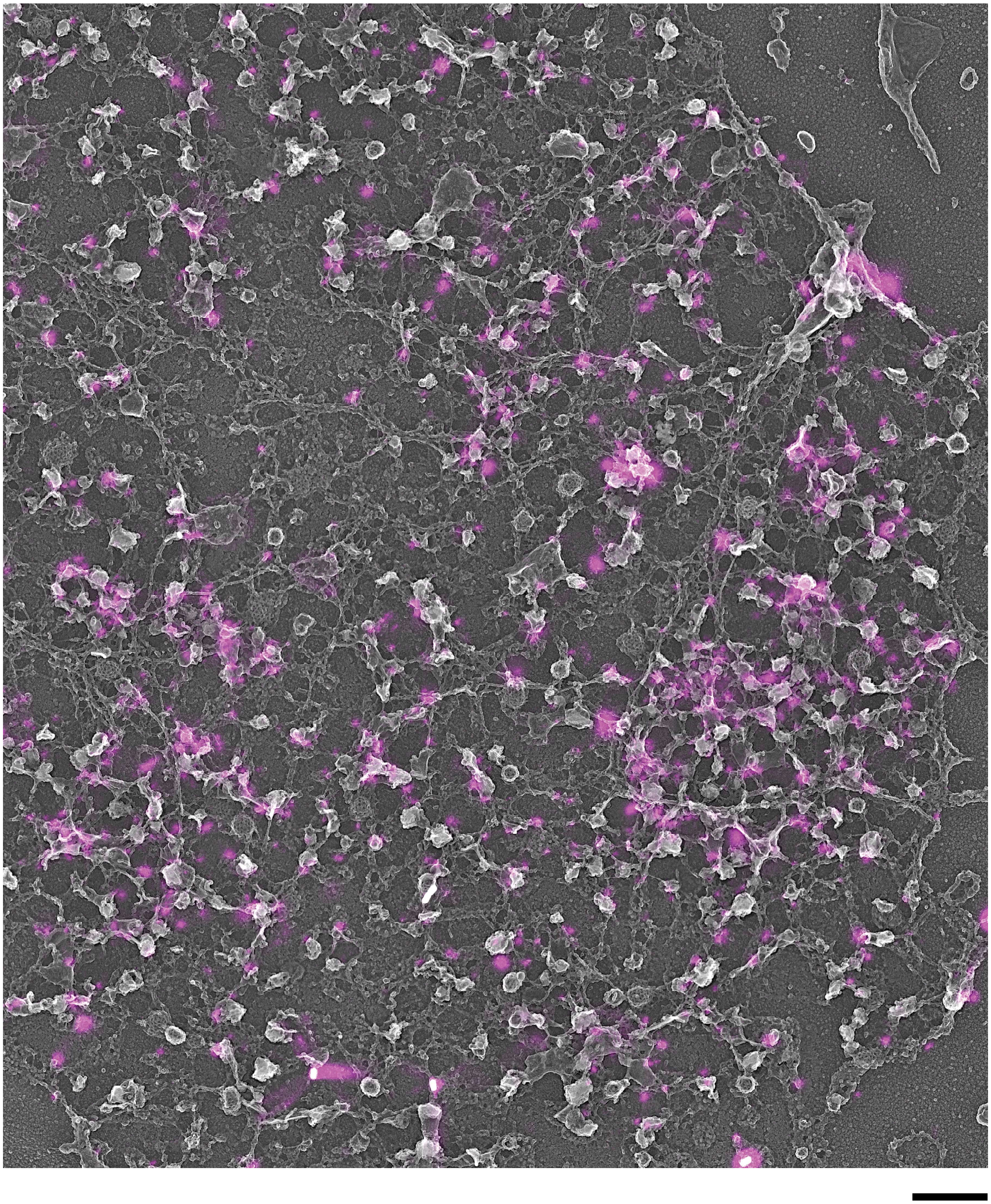

Supplementary Figure 11 continued

Anti-Rab3a

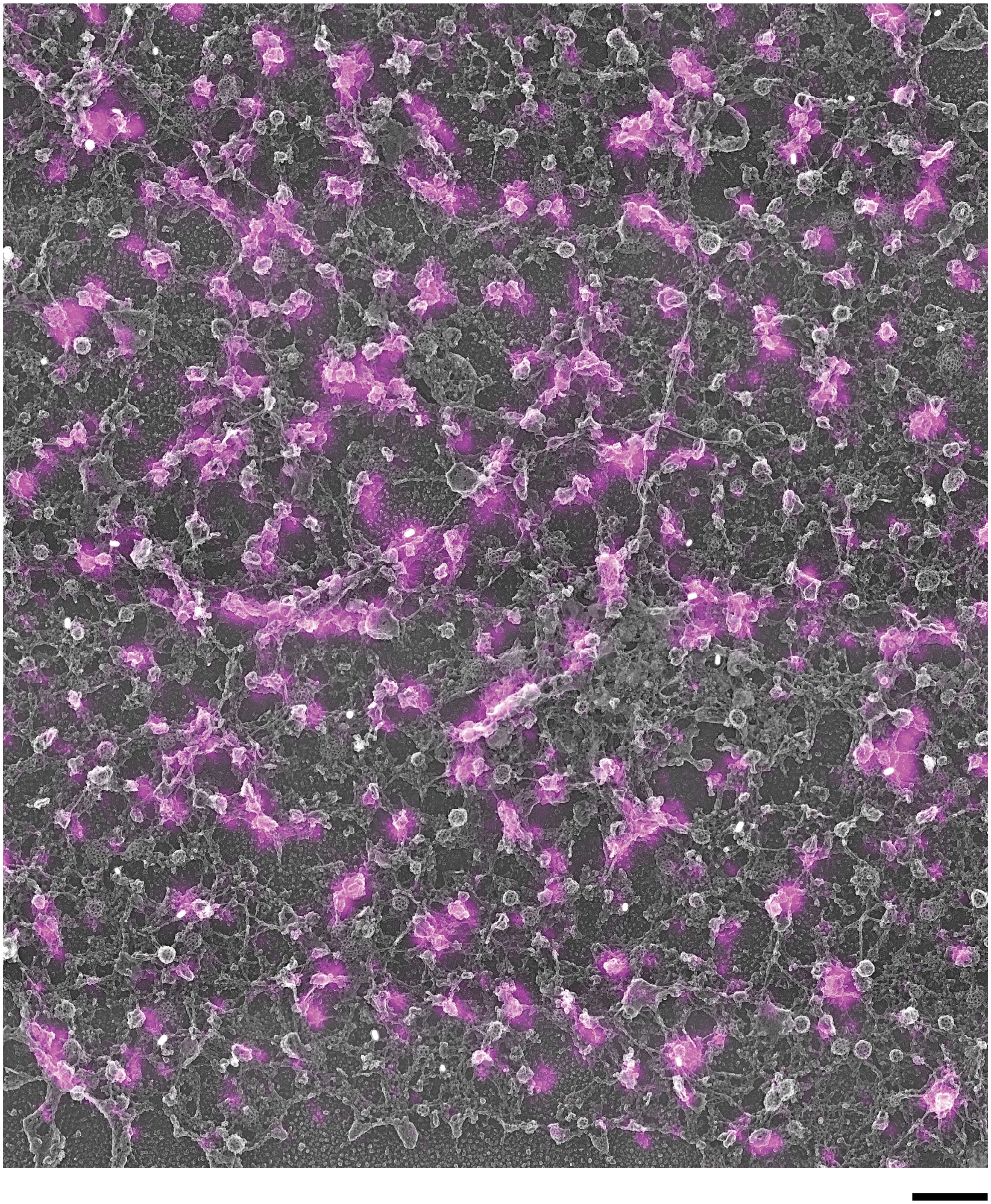

Supplementary Figure 11 Continued

Anti-Granuphilin-a

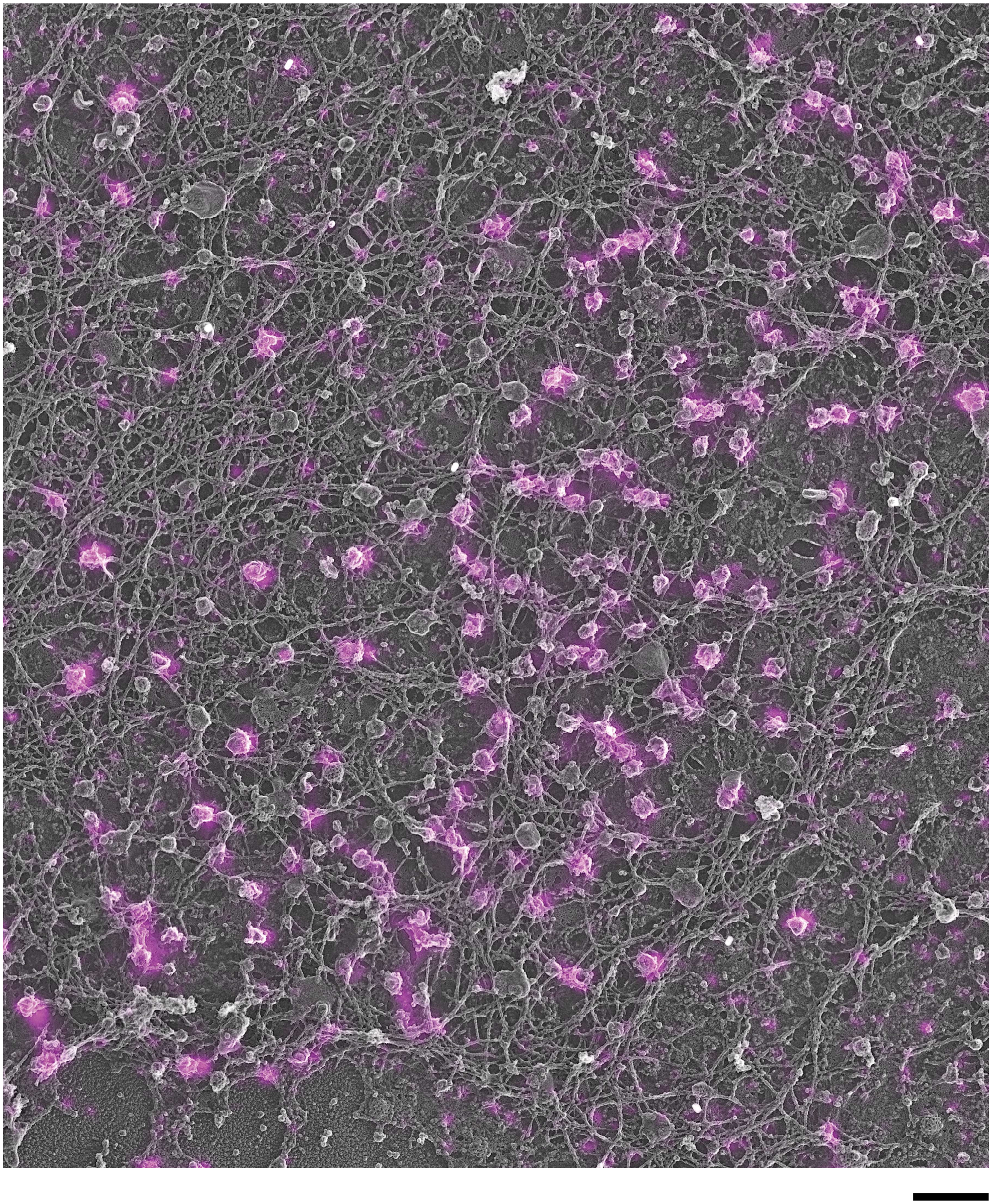

Supplementary Figure 11. Original correlative STORM and TEM images of cells from which the cropped images in Figure 2 were derived. CLEM image for GFP-Rab3a, GFP-Rab27a, GFP-Rab-philin3a, GFP-Granuphilin-a, and immunolabeled Rim2, Rab3a, and Granuphilin-a in PC12 cells. Scale bars are 500 nm. Overexpression CLEM data were collected from two independent experiments and immunolabeled CLEM data from one experiment.

Supplementary Figure 11 Continued

Supplementary Figure 12

His-GFP-Clathrin Light Chain A (Large membrane image for crops in Fig. 3)

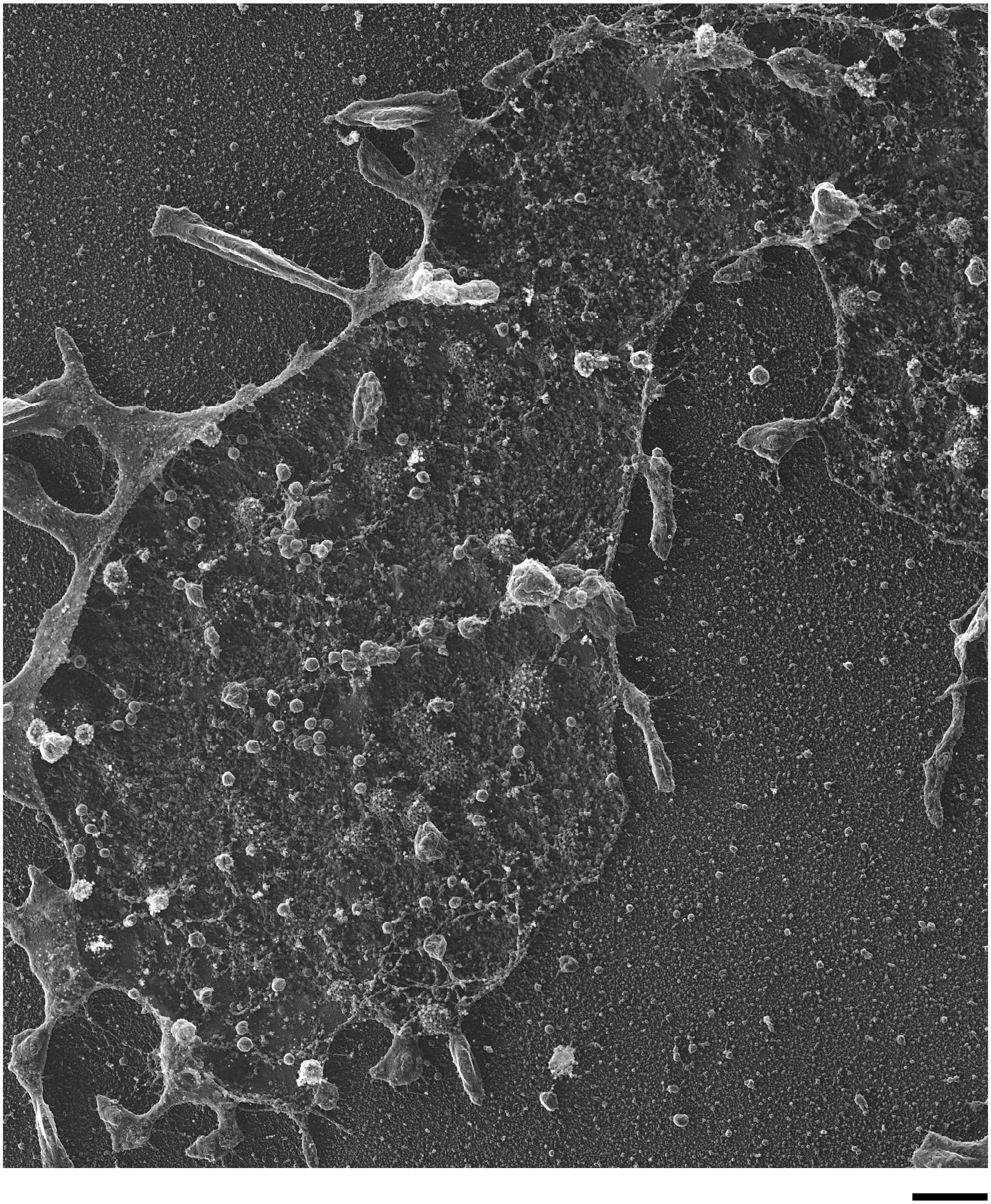

EPS15-GFP-His (Large membrane image for crops in Fig. 3)

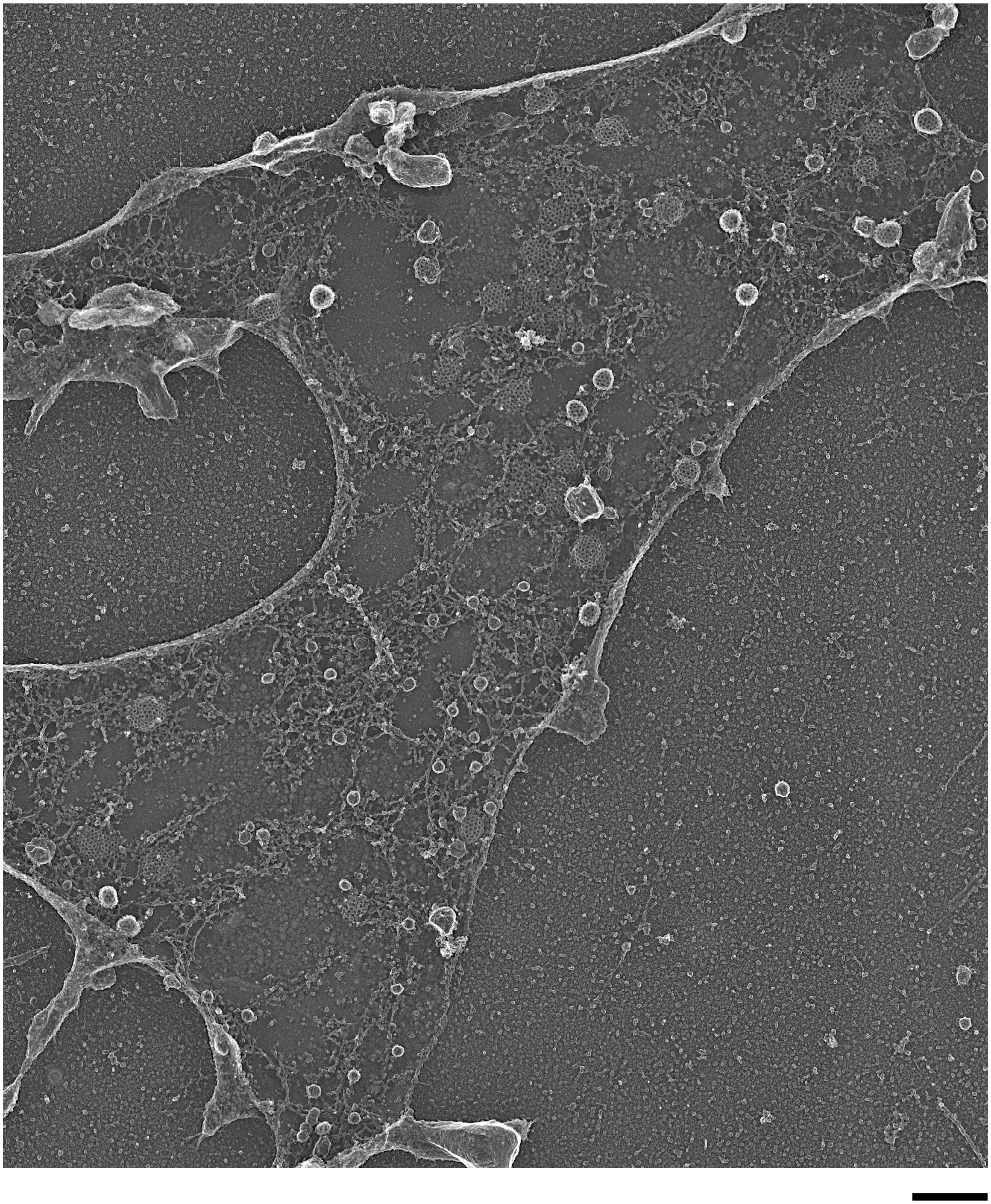

Supplementary Figure 12 Continued

His-Cavin-GFP (Large membrane image for crops in Fig. 3)

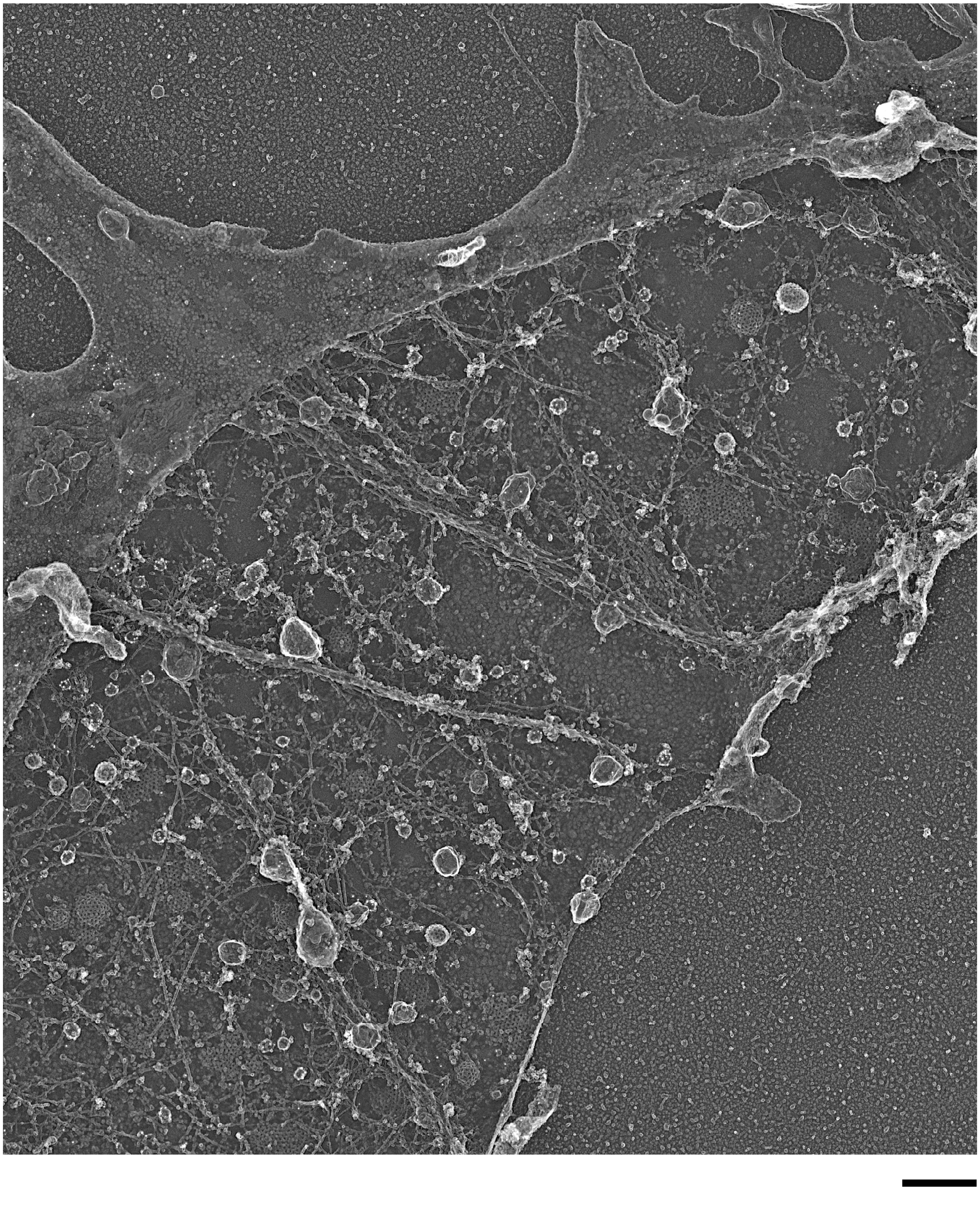

Supplementary Figure 12 Continued

His-GFP-Rab3a (Large image for crops in Fig. 4)

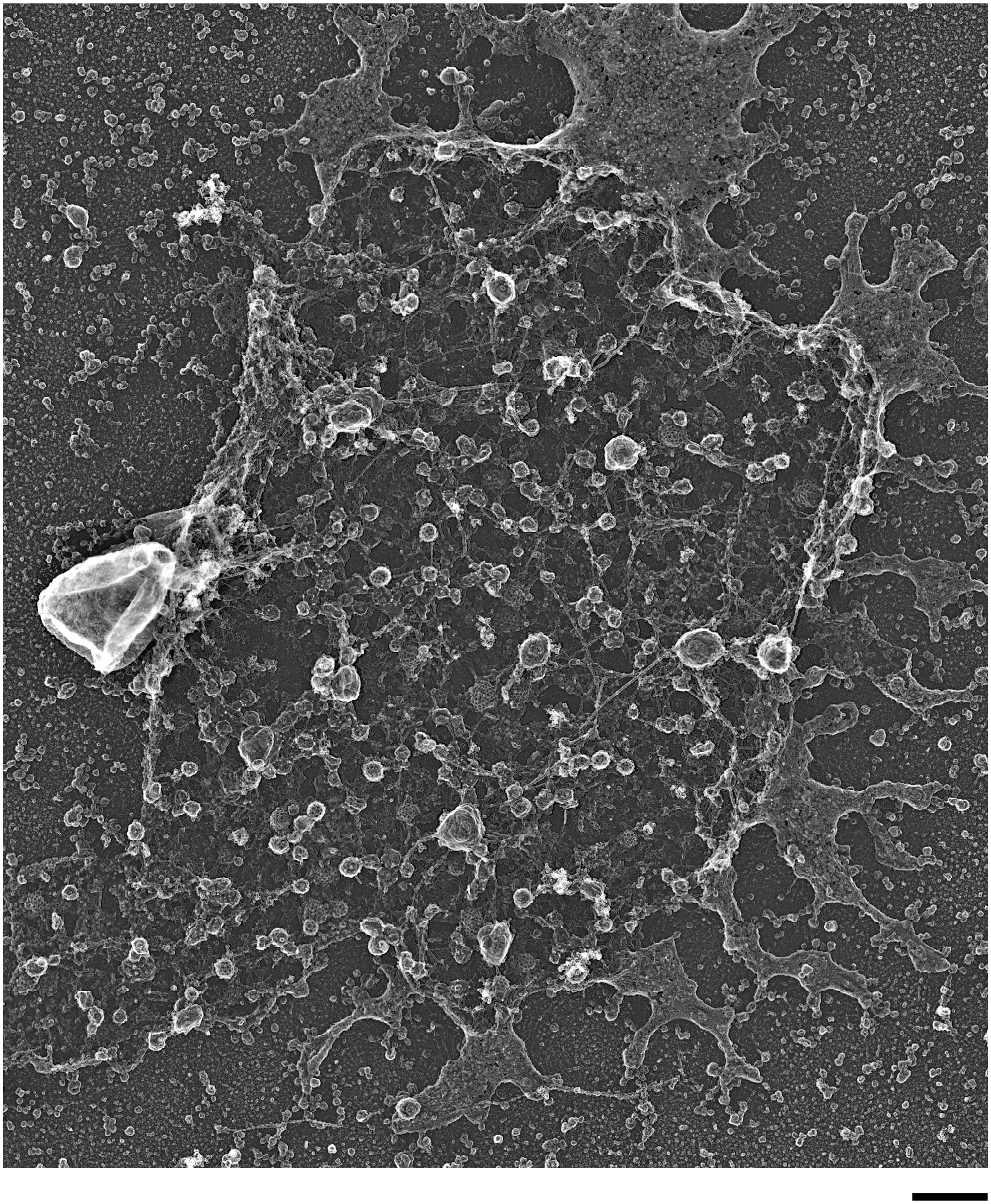

Supplementary Figure 12 Continued

His-GFP-Rab27a (Large image for crops in Fig. 4)

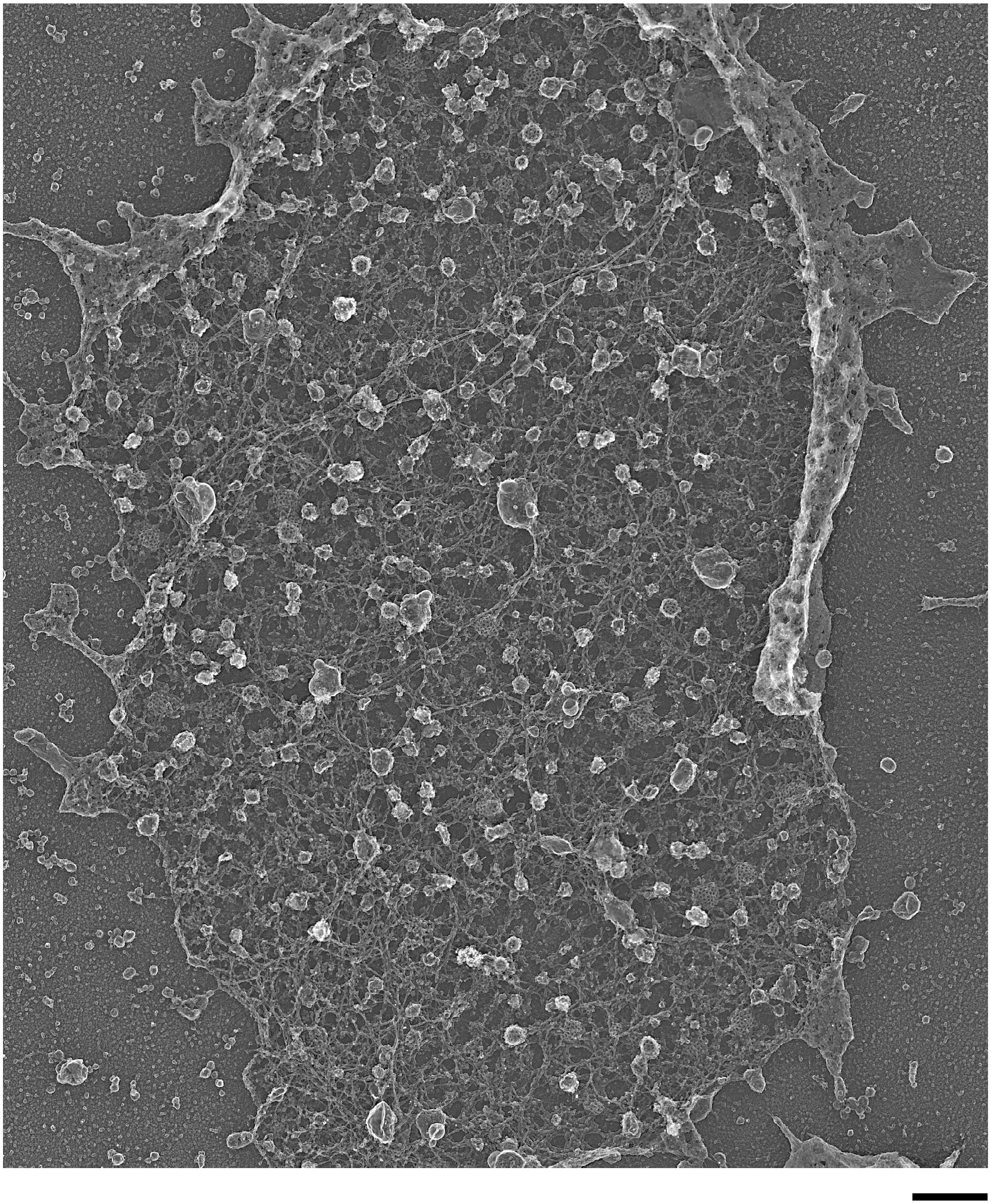

Supplementary Figure 12 Continued

His-GFP-Rabphilin3a (Large image for crops in Fig. 4)

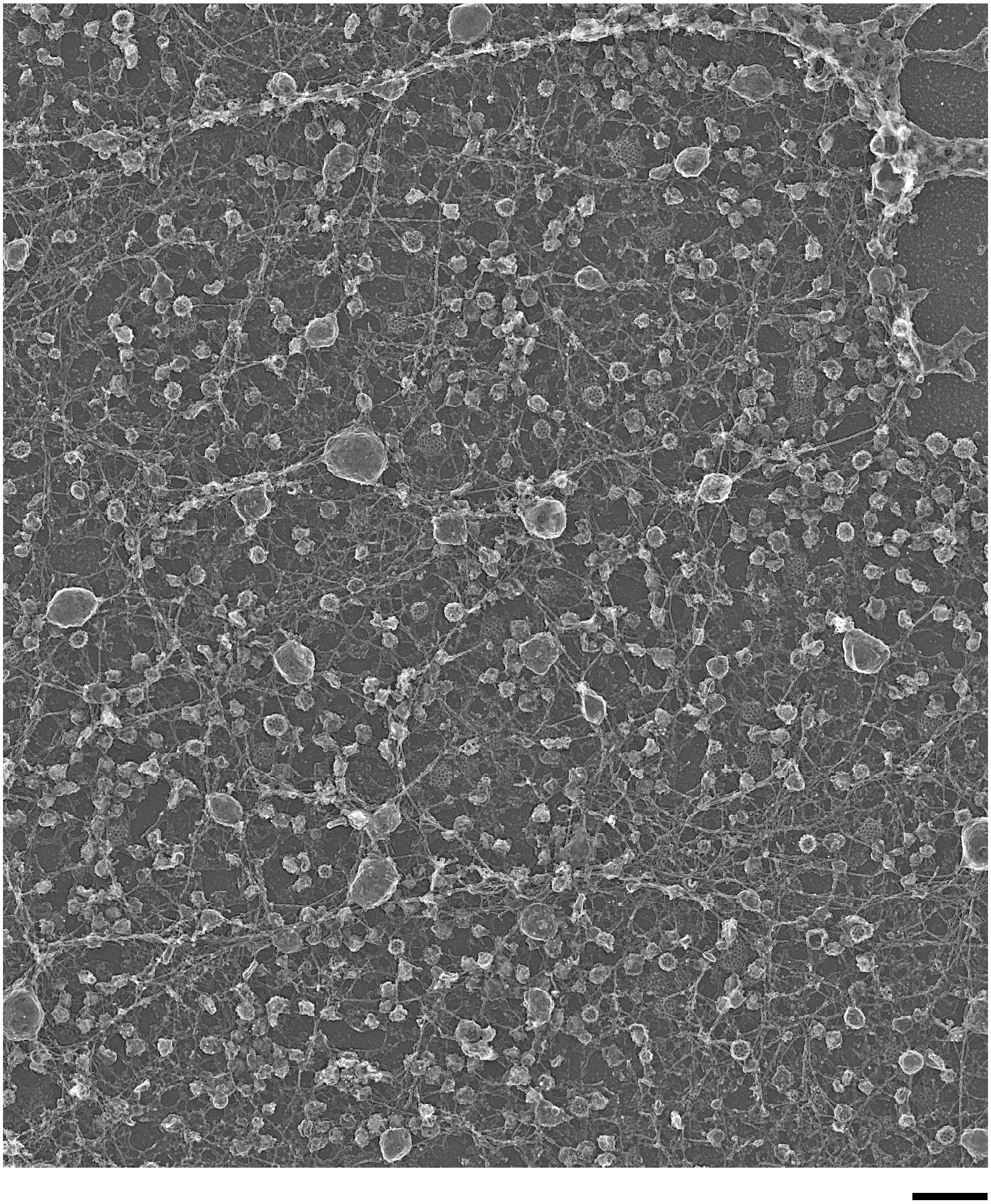

Supplementary Figure 12 Continued

His-GFP-Granuphilin-a (Large image for crops in Fig. 4)

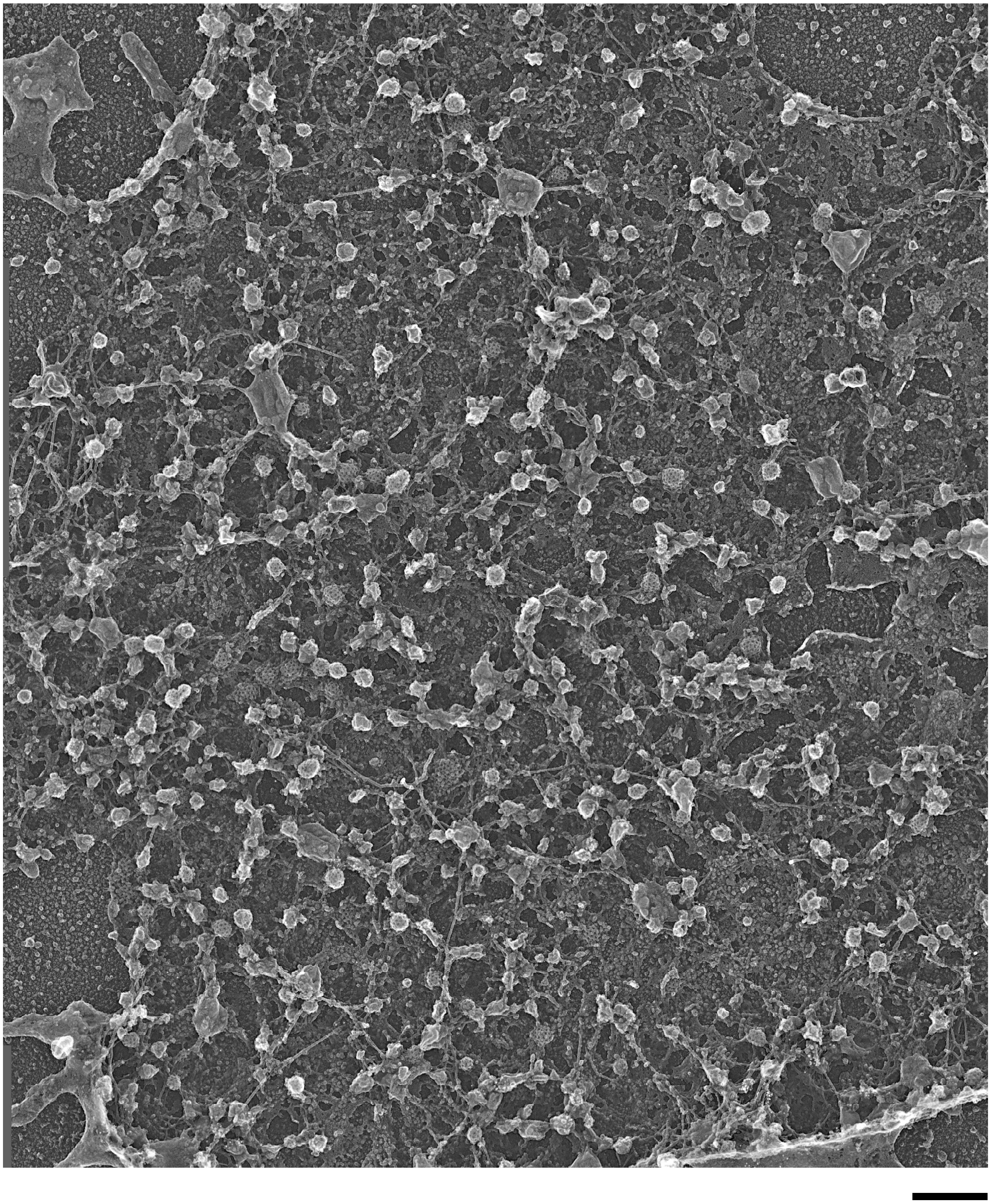

Supplementary Figure 12 Continued

His-GFP-Rim2 (Large image for crops in Fig. 4)

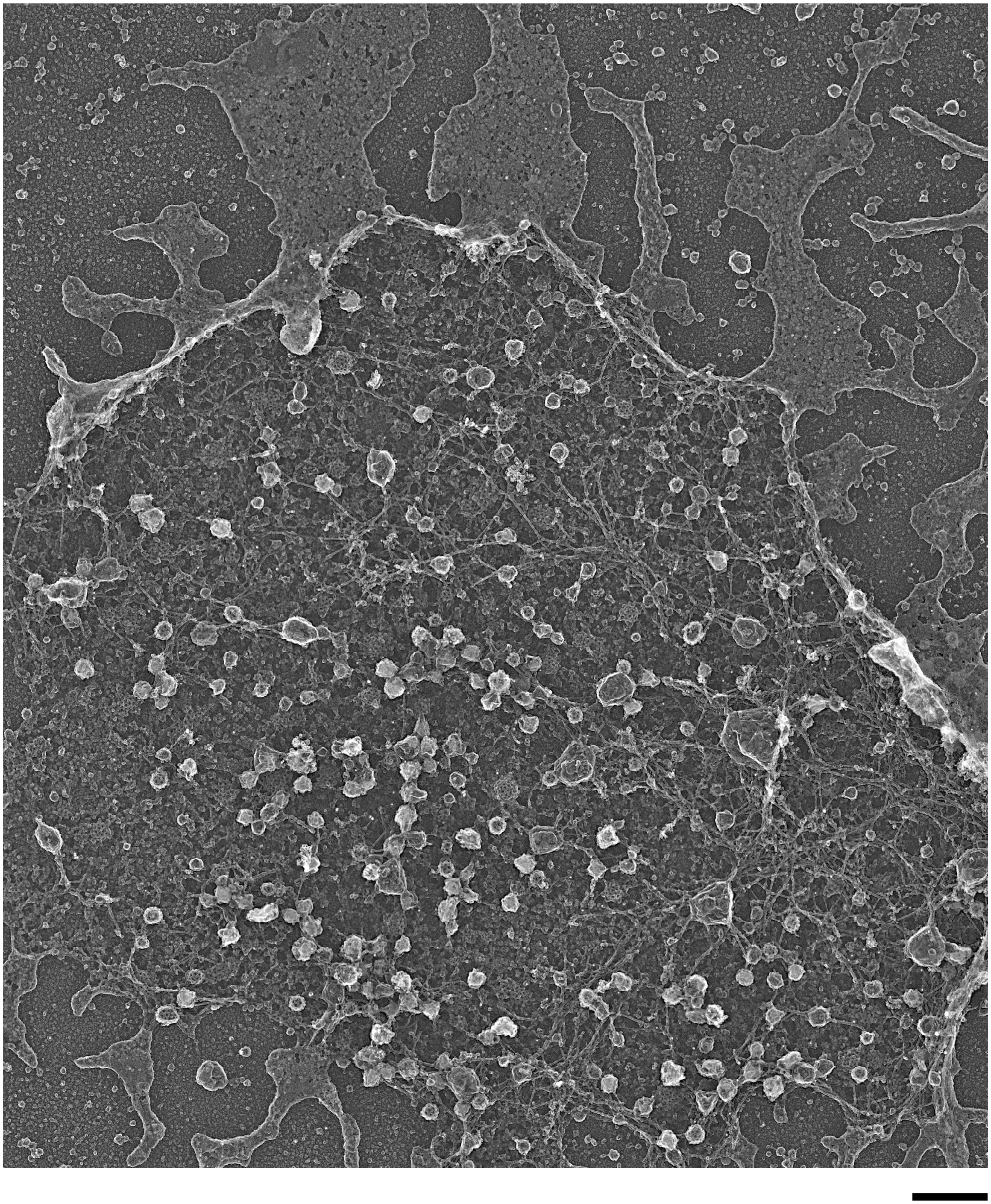

Supplementary Figure 12 Continued

His-GFP-Syntaxin1A (Large membrane image for crops in Fig. 6)

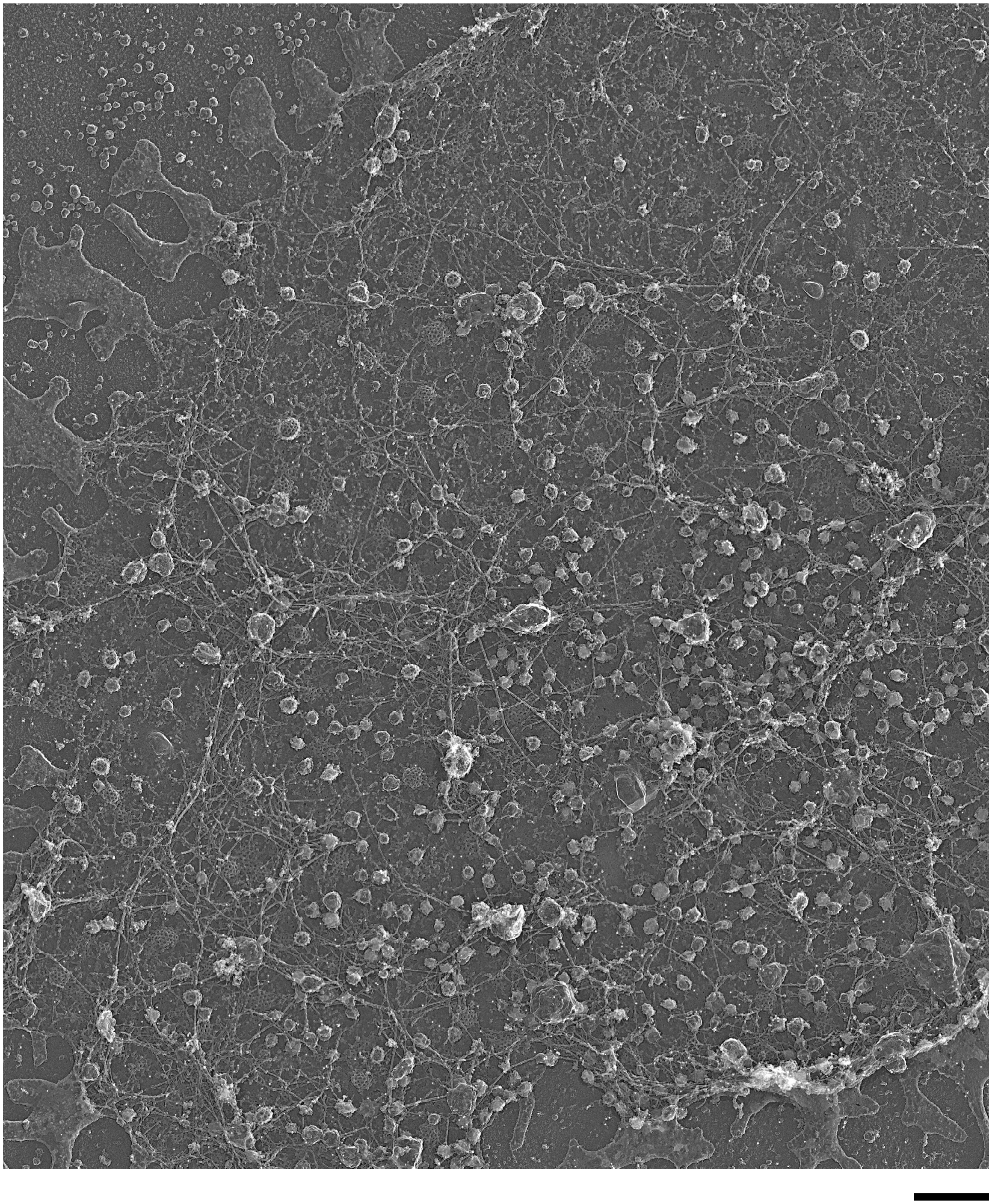

Supplementary Figure 12 Continued

His-GFP-SNAP25 (Large membrane image for crops in Fig. 6)

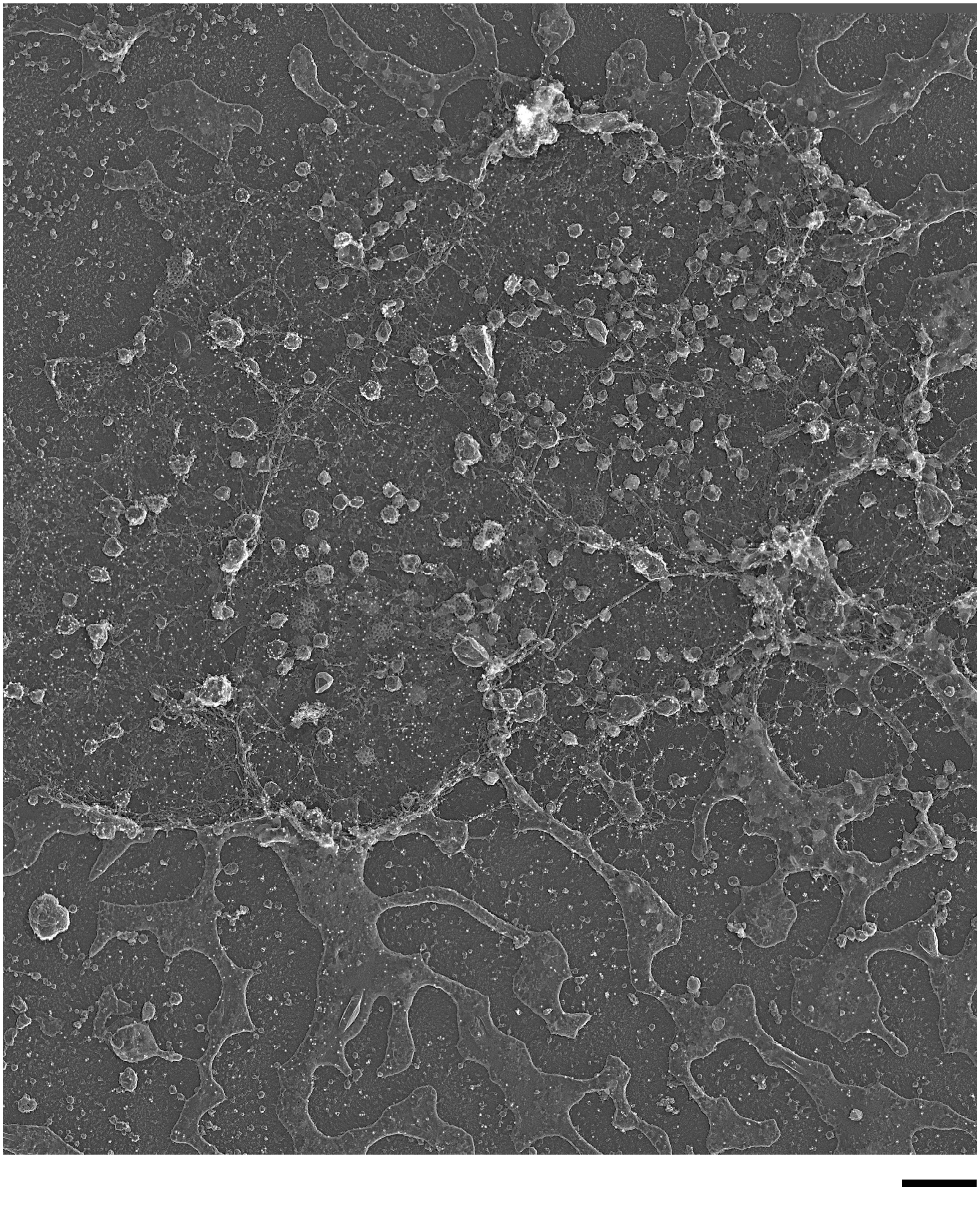

Supplementary Figure 12 Continued

His-GFP-Clathrin Light Chain A (U87-MG)

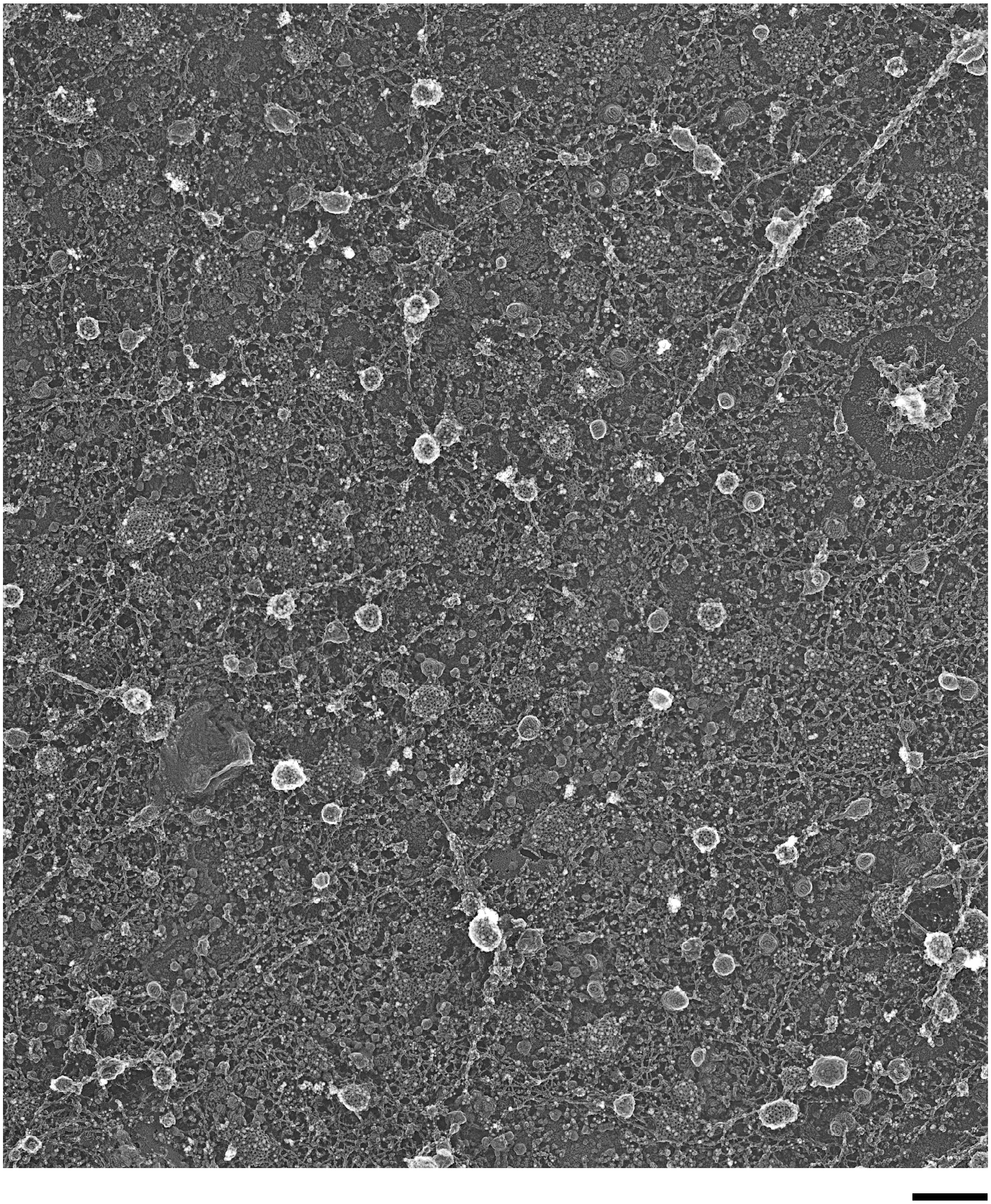

Supplementary Figure 12 Continued

His-GFP-FCHO2 (Hela)

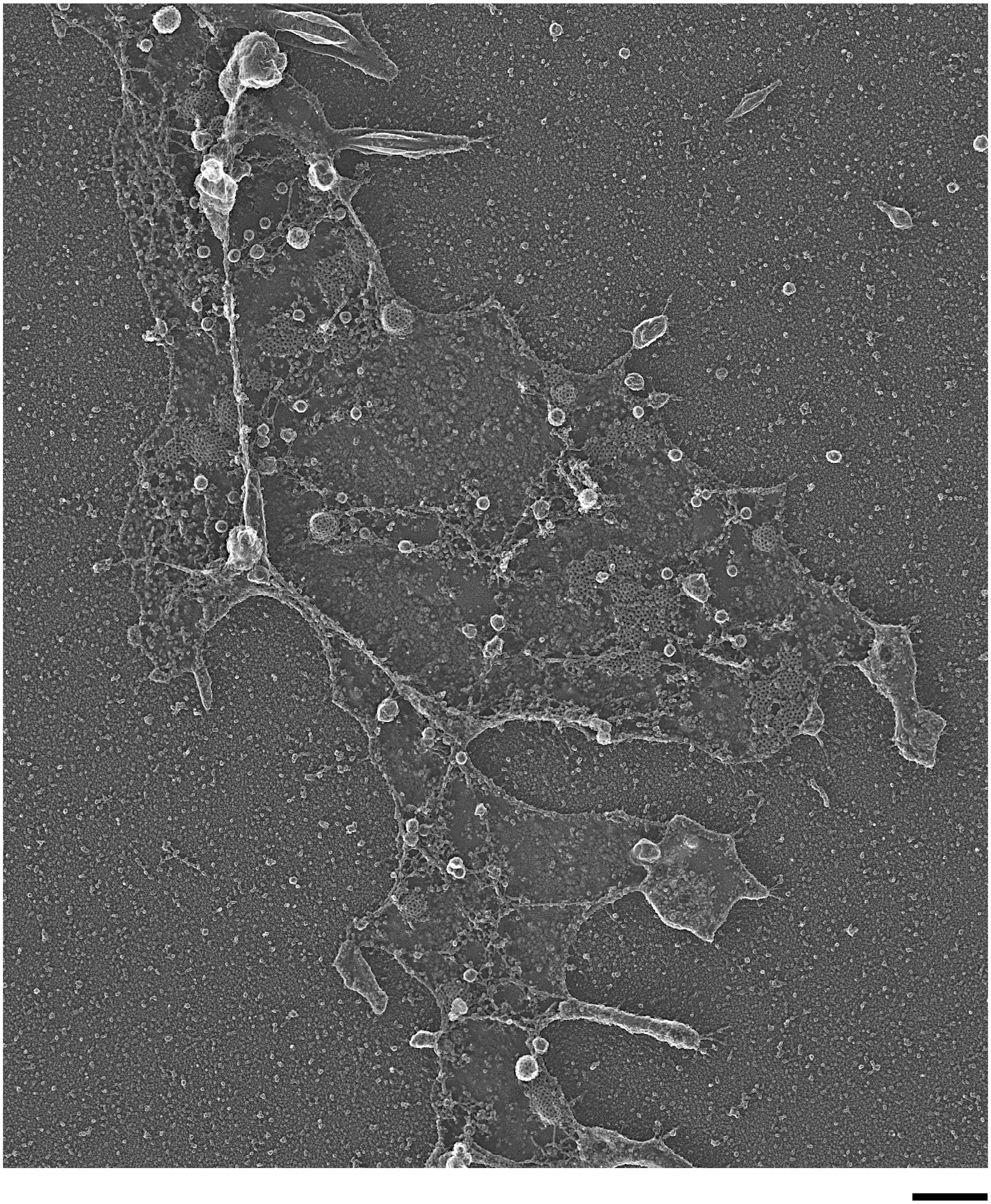

Supplementary Figure 12 Continued

His-GFP-Rab27a-INS-1

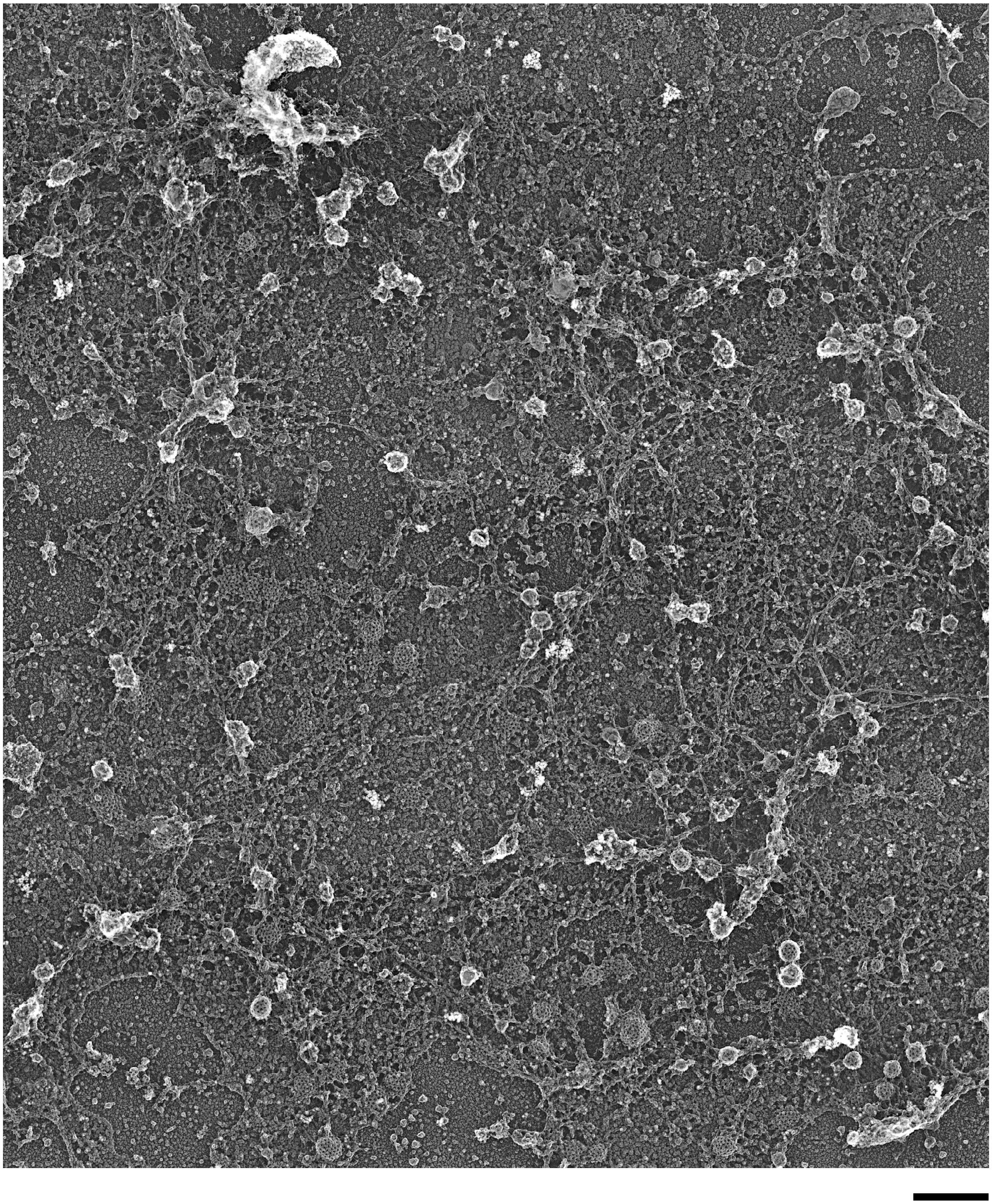

Supplementary Figure 12. Original platinum replica TEM images of cells from which the cropped images in Figure 3 and 4 were derived. PREM images for cells transfected with His-GFP-clathrin light chain A, His-Cavin1-GFP, EPS15-GFP-His, His-GFP- Rab3a, His-GFP- Rab27a, His-GFP- Rabphilin3a, His-GFP- Granuphilin-a, His-GFP-Rim2, His-GFP-Syntaxin1A, and His-GFP-SNAP25, and labeled with Ni-NTA-Au. PREM images for gold labeled U87-MG expressed with His-GFP-clathrin light chain A, Hela cells expressed with His-GFP-FCHO2, and INS-1 cells expressed with His-GFP-Rab27a are also shown. Scale bars are 500 nm. Two independent imaging experiments were performed except for Hela expressing His-GFP-FCHO2, U87-MG expressing His-GFP-clathrin light chain A, and INS-1 expressing His-GFP-Rab27a.

Supplementary Figure 12 Continued

Supplementary Table 1. Table of samples used in the fluorescence profile and radii analysis. **(a)** The table shows the number of cells, coverslips, and dense core vesicles used in the analysis of dark GFP fusion proteins, Rab3a, Rab27a, Rabphilin3a, Granuphilin-a, and for immunolabeled Rab3a, Granuphilin-a, and Rim2. **(b)** The number of vesicles analyzed for radial and edge profiles are listed separately.The table shows the number of vesicles used in vesicle radii analysis.

Table 1a

Table of the number of coverslip, cells, and vesicles analyzed to generate fluorescence profile

	Cell N	Coverslip N	Vesicles N(radial)	Vesicles N(edge)
dGFP-Rab3a	8	2	458	455
dGFP-Rab27a	7	3	545	544
dGFP-Rabphilin3a	8	3	644	659
dGFP-Granuphilina	10	2	275	285
Rab3a-Ig	7	1	596	598
Granuphilin-a-Ig	2	1	252	252
Rim2-Ig	5	1	129	128
sum	47	13	2899	2921

Table 1b

Table of number of cells and vesicles analyzed for vesicle radii

	Cell N	Vesicles
dGFP-Rab3a	8	451
dGFP-Rab27a	7	514
dGFP-Rabphilin3a	8	674
dGFP-Granuphilina	10	284
Rab3a-Ig	7	581
Granuphilin-a-Ig	2	244
Rim2-Ig	5	126
Untransfected cell (PREM)	3	521
Untransfected cell (ultra-thin TEM)	8	118

Supplementary Table 2. Table of number of cells, vesicles, and gold nanoparticles used in the tomogram analysis. The table shows types of cells used, number of tomograms, vesicles, and gold particles analyzed from two independent experiments and the combined data for His-GFP-ClCa, Cavin-GFP-His, EPS15-GFP-His, His-GFP-Rab27a, His-GFP-Rab3a, His-GFP-Granuphilin-a, His-GFP-Rabphilin3a, His-GFP-Rim2, His-GFP-Syntaxin1A, and His-GFP-SNAP25 overexpressed

**EXPT#1**						
**Cell Type**	**Tomogram #**	**His fusion(N-term)**	**Protein**	**His fusion(C-term)**	**vesicle #**	**GNP #**
Hela	4	His_GFP-	Clathrin Light ChainA		22	320
Hela	3	His_Cavin1	Cavin1	GFP	30	331
Hela	4		EPS15	GFP_His	36	128
PC12	2	His_GFP	Rab27a		27	198
PC12	3	His_GFP	Rab3a		29	335
PC12	3	His_GFP	Granuphilin-a		30	257
PC12	4	His_GFP	Rabphilin3a		31	276
PC12	4	His_GFP	Rim2		31	303
PC12	4	His_GFP	Syntaxin1a		31	135
PC12	3	His_GFP	SNAP25		35	209
**EXPT#2**						
Hela	4	His_GFP-	Clathrin Light ChainA		32	380
Hela	3	His_	Cavin1	GFP	33	226
Hela	5		EPS15	GFP-His	29	65
PC12	3	His_GFP	Rab27a		31	265
PC12	2	His_GFP	Rab3a		30	249
PC12	3	His_GFP	Granuphilina		32	278
PC12	3	His_GFP	Rabphilin3a		27	179
PC12	7	His_GFP	Rim2		32	211
PC12	5	His_GFP	Syntaxin1a		45	152
PC12	4	His_GFP	SNAP25		30	139
	**# of Tomagrams**				**# of vesicles**	**# of GNP**
Sum	73				623	4636

Expt 1 and 2

		**Total cell #**	**Total vesicle #**	**Total GNP #**
Hela	Clathrin Light ChainA	8	54	700
Hela	Cavin1	6	63	557
Hela	EPS15	9	65	193
PC12	Rab27a	5	58	463
PC12	Rab3a	5	59	584
PC12	Granuphilin-a	6	62	535
PC12	Rabphilin3a	7	58	455
PC12	Rim2	11	63	514
PC12	Syntaxin1a	9	76	287
PC12	SNAP25	7	65	348

Supplementary Table 3. Table of information about plasmids used in our study. The table includes the sequenced-confirmed plasmids used in CLEM experiments and histidine based protein labeling with Ni-NTA-Au.

Plasmid name	N-terminal protein	C-terminal protein
dGFP-Rab27a	dark GFP	*Homo sapiens* RAB27A, member RAS oncogene family (Rab27A)
dGFP-Rab3a	dark GFP	*Mus musculus* RAB3A, member RAS oncogene family (Rab3a)
dGFP-Rabphilin3a	dark GFP	*Bos taurus* rabphilin 3A homolog (mouse)
dGFP-Granuphilin-a	dark GFP	*Homo sapiens* synaptotagmin-like 4
His-GFP-Clathrin	Histidine & EGFP	*Mus musculus* clathrin, light polypeptide (Lca)
His-Cavin1-GFP	*Mus musculus* caveolae associated 1 (Cavin1)	EGFP Cloning vector ENCODE_CRISPR_GFP GFP tag fusion protein gene
EPS15-GFP-His	*Homo sapiens* epidermal growth factor receptor pathway substrate 15 (EPS15)	monomeric EGFP (A206K)
His-GFP-Rab27a	Histidine & EGFP	*Homo sapiens* RAB27A, member RAS oncogene family (Rab27A)
His-GFP-Rab3a	Histidine & EGFP	(Rab3a)
His-GFP-Rabphilin	Histidine & EGFP	*Bos tauru* s rabphilin 3A homolog (mouse)
His-GFP-Granuphilir	Histidine & EGFP	*Homo sapiens* synaptotagmin-like 4
His-GFP-Rim2	Histidine & EGFP	*Rattus norvegicus* RIM2
His-GFP-Syntaxin1A	Histidine & EGFP	*Rattus norvegicus* syntaxin 1A (Stx1a)
His-GFP-SNAP25	Histidine & EGFP	*Mus musculus* synaptosomal-associated protein 25 (Snap25)

Supplementary Table 4. Table showing primers used in the cloning of His-tagged proteins of interests.

Forward/Rev	Plasmid template	Primer Sequence	Insertion
Forward	*GFP-POI	CAT CAT CAC AGC AGC GGC GTG AGC AAG GGC GAG GAG	6x His
Reverse		ATG ATG ATG GCT GCT GCC CAT GGT GGC GAC CGG TAG	
Forward	*Cavin1-GFP	CCACAGCAGCGGCGAGGATGTCACGCTCCAT	6x His
Reverse		TGGTGATGATGATGCATGGCGAAGCTTAAGTTTAAAC	
Forward	*GFP-Rim2	GTGAGCAAGGGCGAGGAG	6x His
Reverse		CATGGTGGCGACCGGTAG	
Forward	EPS15-GFP*	TCATCATCATCACTAAAGCGGCCGCGACTCT	6x His
Reverse		TGATGGCTGCTGCCCTTGTACAGCTCGTCCATGC	

POI: His-GFP-Clathrin Light Chain A, His-GFP-Rab27a, His-GFP-Rab3a, His-GFP-Rabphilin3a, His-GFP-Granuphilin-a, His-GFP-SNAP25, and His-GFP-Syntaxin1A.

* indicates the terminal where 6X-Histidine is tagged.
