# Supplementary material for: The nanoscale molecular morphology of docked exocytic dense-core vesicles in neuroendocrine cells
Source: Nat Commun. 2021 Jun 25;12:3970. doi: 10.1038/s41467-021-24167-9 (PMC8233335; doi:10.1038/s41467-021-24167-9)
Supplement: Supplementary file 3 — Description of Additional Supplementary Files [file 41467_2021_24167_MOESM3_ESM.pdf]

Description of Additional Supplementary Files

File Name: Supplementary Movie 1
Description: Tomogram of a Hela cell labeled with Ni-NTA-Au against His-GFP-clathrin light chain A. The tomogram is viewed in the XY plane through Z slices. Each voxel is 2.3 nm in all dimensions. The tomogram is 2.4 mm in width and 1.9 mm in height.

File Name: Supplementary Movie 2
Description: Tomogram of a Hela cell labeled with Ni-NTA-Au against His-cavin1-GFP. The tomogram is viewed in the XY plane through Z slices. Each voxel is 2.3 nm in all dimensions. The tomogram is 2.4 mm in width and 1.9 mm in height.

File Name: Supplementary Movie 3
Description: Tomogram of a Hela cell labeled with Ni-NTA-Au against EPS15-GFP-His. The tomogram is viewed in the XY plane through Z slices. Each voxel is 2.3 nm in all dimensions. The tomogram is 2.4 mm in width and 1.9 mm in height.

File Name: Supplementary Movie 4
Description: Tomogram of a PC12 cell labeled with Ni-NTA-Au against His-GFP-Rab27a. The tomogram is viewed in the XY plane through Z slices. Each voxel is 2.3 nm in all dimensions. The tomogram is 2.3 mm in width and 1.9 mm in height.

File Name: Supplementary Movie 5
Description: Tomogram of a PC12 cell labeled with Ni-NTA-Au against His-GFP-Rab3a. The tomogram is viewed in the XY plane through Z slices. Each voxel is 2.3 nm in all dimensions. The tomogram is 1.6 mm in width and 1.8 mm in height.

File Name: Supplementary Movie 6
Description: Tomogram of a PC12 cell labeled with Ni-NTA-Au against His-GFP-Granuphilin-a. The tomogram is viewed in the XY plane through Z slices. Each voxel is 2.3 nm in all dimensions. The tomogram is 2.3 mm in width and 1.9 mm in height.

File Name: Supplementary Movie 7
Description: Tomogram of a PC12 cell labeled with Ni-NTA-Au against His-GFP-Rabphilin3a. The tomogram is viewed in the XY plane through Z slices. Each voxel is 2.3 nm in all dimensions. The tomogram is 2.3 mm in width and 1.9 mm in height.

File Name: Supplementary Movie 8
Description: Tomogram of a PC12 cell labeled with Ni-NTA-Au against His-GFP-Rim2. The tomogram is viewed in the XY plane through Z slices. Each voxel is 2.3 nm in all dimensions. The tomogram is 2.9 mm in width and 1.9 mm in height.

File Name: Supplementary Movie 9
Description: Tomogram of a PC12 cell labeled with Ni-NTA-Au against His-GFP-Syntaxin1A. The tomogram is viewed in the XY plane through Z slices. Each voxel is 2.3 nm in all dimensions. The tomogram is 2.3 mm in width and 1.9 mm in height.

File Name: Supplementary Movie 10
Description: Tomogram of a PC12 cell labeled with Ni-NTA-Au against His-GFP-SNAP25. The tomogram is viewed in the XY plane through Z slices. Each voxel is 2.3 nm in all dimensions. The tomogram is 2.3 mm in width and 1.9 mm in height.

File Name: Supplementary Movie 11
Description: Video of 500 frames of raw localization imaging shown by fluorescence blinks.
